# A Modular Cloning Toolkit Including CRISPRi for the Engineering of the Human Fungal Pathogen and Biotechnology Host *Candida glabrata*

**DOI:** 10.1021/acssynbio.2c00560

**Published:** 2023-04-12

**Authors:** Sonja Billerbeck, Rianne C. Prins, Malte Marquardt

**Affiliations:** † Department for Molecular Microbiology, Groningen Biomolecular Sciences and Biotechnology Institute, University of Groningen, Nijenborgh 7, 9747 AG Groningen, The Netherlands

**Keywords:** *Candida glabrata*, modular cloning toolkit, Golden Gate cloning, CRISPR interference

## Abstract

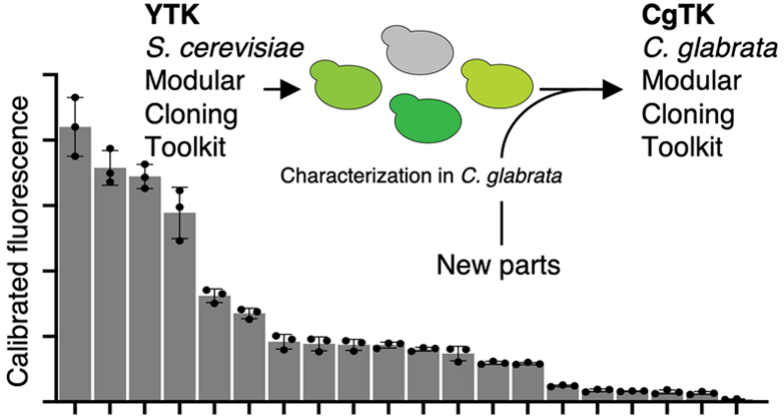

The yeast *Candida glabrata* is an emerging, often drug-resistant opportunistic human pathogen that can cause severe systemic infections in immunocompromised individuals. At the same time, it is a valuable biotechnology host that naturally accumulates high levels of pyruvate—a valuable chemical precursor. Tools for the facile engineering of this yeast could greatly accelerate studies on its pathogenicity and its optimization for biotechnology. While a few tools for plasmid-based expression and genome engineering have been developed, there is no well-characterized cloning toolkit that would allow the modular assembly of pathways or genetic circuits. Here, by characterizing the *Saccharomyces cerevisiae*-based yeast molecular cloning toolkit (YTK) in *C. glabrata* and by adding missing components, we build a well-characterized CgTK (*C. glabrata* toolkit). We used the CgTK to build a CRISPR interference system for *C. glabrata* that can be used to generate selectable phenotypes via single-gRNA targeting such as is required for genome-wide library screens.

## Introduction

*Candida glabrata* is an opportunistic human fungal pathogen, causing 10–25% of fungal bloodstream infections in humans.^1−5^ Clinical isolates are increasingly resistant to multiple drugs, and as a consequence, mortality rates of blood-stream infected individuals are high.^6−9^ Unlike other fungal pathogens that are acquired from the environment, *C. glabrata* is a natural commensal of the human mycobiome, mostly the GI tract,^1,10,11^ but it can overgrow and turn virulent once the immune system of the human host is compromised. The virulent phenotype is linked to *C. glabrata*’s ability to grow rapidly at 37 °C, its high capacity for adhesion and biofilm formation, its intrinsic ability to tolerate certain antifungal drugs, and its rapid adaptation to stresses.^8,12−14^ Although much progress has been made in understanding *C. glabrata*’s biology, the molecular regulatory underpinnings that enable the phenotypic adaptation to a pathogenic lifestyle are not yet understood.

At the same time, some *C. glabrata* strains are used as valuable chassis for biotechnology as they naturally produce high levels of pyruvic acid^15,16^—an important precursor for agrochemistry and a dietary supplement—and they have been engineered to metabolize pyruvic acid into valuable downstream products such as α-ketoglutarate,^17^ fumarate,^18^ acetoin,^19^ malate,^20^ and diacetyl.^21^

Tools enabling a synthetic biology approach to *C. glabrata*’s biology could not only facilitate its metabolic engineering toward biotechnological applications but also help elucidate the genetic and regulatory architectures underlying the phenotypic transition to virulence.^22^ In addition, understanding the genetic design of this transition could become a valuable inspiration for Synthetic Biology and help design sophisticated programmed cellular behavior.

Some genetic tools exist for *C. glabrata*, such as a set of expression plasmids,^23^ several nonhomologous-end-joining-based CRISPR tools,^24,25^ as well as transposons for gene disruption^26,27^ and a deletion collection covering 12% of the *C. glabrata* genome.^28^ Here, we extend the genetic toolbox for *C. glabrata* by building a molecular cloning toolkit (CgTK) by recharacterizing and extending the Yeast Toolkit (YTK) built for *Saccharomyces cerevisiae.* Modular cloning kits based on Golden Gate assembly^29^ have proven indispensable to overcome cloning barriers for bacterial and eukaryotic hosts, including plants.^30−33^

## Results and Discussion

### Design of the *Candida glabrata* Toolkit (CgTK)

Given the close relationship between *S. cerevisiae* and *C. glabrata*,^12^ we reasoned that the most resource-saving way to build the CgTK was to use the *S. cerevisiae* YTK and characterize part performance in *C. glabrata*, identify malperforming parts, and add missing ones. In total, we characterized 21 constitutive promoters (19 YTK, 2 new), four constitutive synthetic minimal promoters (4 new), three inducible promoters (1 YTK, 2 new), and three protein degradation tags (3 YTK). We constructed eight vector sets for the assembly of (multiple) transcriptional units featuring four auxotrophic markers and two origins of replication and we built a CRISPRi system (Supplementary Figure S1 and Supplementary Tables S1 and S2). We used *C. glabrata* ATCC 2001 HTL^–28^ as a host, a clinical isolate frequently used for research including for the creation of the *C. glabrata* deletion collection.^28^

### A Set of Cloning Vectors

First, we created an eight-membered green-white screening compatible vector set featuring four selection markers combined with either the YTK*-*derived ScCEN/ARS origin of replication from *S. cerevisiae* or a *C. glabrata* derived CgCEN/ARS. Most *C. glabrata* research relies on plasmids encoding the CgCEN/ARS, while most available yeast plasmids (e.g., on Addgene) contain an ScCEN/ARS. To facilitate the use of plasmids designed for *S. cerevisiae* in *C. glabrata*, we were interested in whether the ScCEN/ARS sequence is functional in *C. glabrata*.

We recorded growth curves to detect potential plasmid burden using “empty” vectors and vectors carrying an HHF1p-Venus-ENO1t transcriptional unit (Supplementary Figures S2 and S3). Vector-specific plasmid burden had been shown before for *S. cerevisiae* and can help in choosing the right vector for an application.^34^ Cells carrying the empty vectors showed overall similar growth behavior on their respective selective media when compared to cells not carrying a vector on nonselective media (Supplementary Figures S2B and C). Only vector version 1.3 caused slightly slowed growth and reduced final OD_630_ (Supplementary Figure S2B). The impact of the *LEU2* marker on growth in *C. glabrata* is consistent with results in *S. cerevisiae*.^34^ In contrast, cells carrying the vector set encoding a transcriptional unit and the ScCEN/ARS showed pronounced growth phenotypes with longer lag phases and lower final OD_630_ (Supplementary Figure S3, Note S1).

### Copy Number Differences Across the Vector Set Allow for Tuning Expression Strength

Given the growth phenotypes, we tested for vector-dependent differences in expression levels and in plasmid copy number: The ScCEN/ARS-based vectors showed a 3- to 8-fold higher expression level when compared to their CgCEN/ARS-based counterparts (Supplementary Figure S4). Relative copy numbers of some of the vectors were compared by qPCR (Supplementary Figure S5) and showed that vector version 1.1 was the most abundant vector, while vector version 2.2 was the least abundant (Supplementary Figure S4A). Our data indicate that not only the origin of replication but also the selection marker seems to impact copy number. Further, copy number alone does not seem to be responsible for growth burden or expression levels: For example, vector 2.1 seems to be as abundant as vector 1.2, but it shows less growth burden (Supplementary Figure S3) but also lower expression (Supplementary Figure S4). These findings are in line with plasmid-growth-burden-related findings for *S. cerevisiae*, supporting the need for good experimental characterization of parts to allow predictable engineering.^34^

### Twenty-One Constitutive Yeast Promoters and Four Synthetic Minimal Promoters

**Figure 1:**
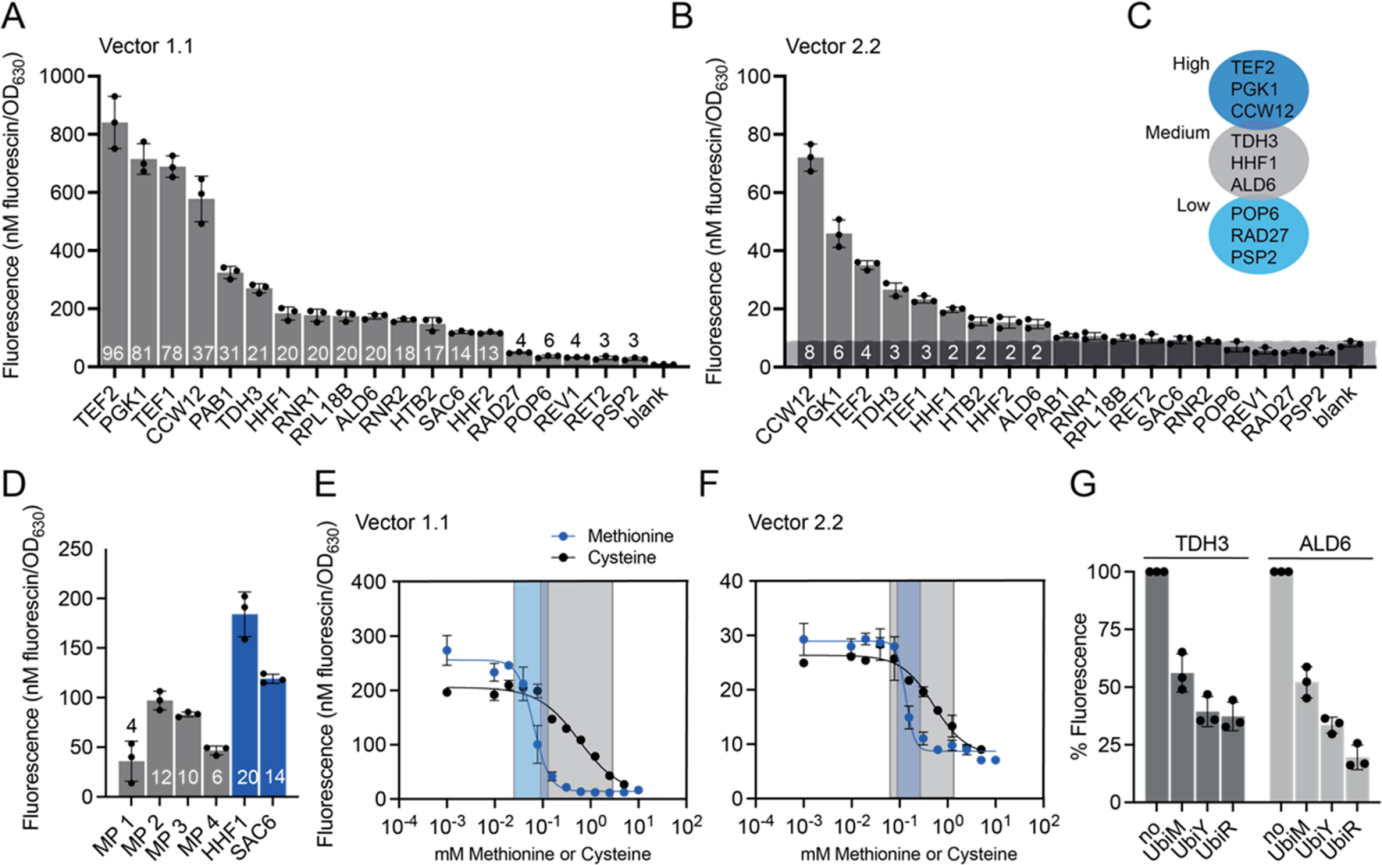
Performance of YTK and additional parts in *C. glabrata* using green fluorescence (Venus) as readout. (A and B) Performance of the 19 YTK promoters in *C. glabrata* using two different vectors: version 1.1 (A) and 2.2 (B) and using the YTK-derives Venus and the ENO1 terminator as readout. The numbers indicate the fold-change in fluorescence over background, where background is the autofluorescence of *C. glabrata* cells. Measurements were taken after 16 h of growth (see Materials and Methods in the SI). Note: The *Y*-axis shows a different scale. Units were calibrated as described in the Materials and Methods. (C) Suggested high, medium, and low expression promoters based on the data displayed in A and B. (D) Performance of minimal yeast promoters in *C. glabrata* using vector version 1.1, promoters are compared to the YTK-derived HHF1p and SAC6p. (E and F) Performance of MET3 promoters in vector version 1.1 (E) and 2.2 (F) when grown in the presence of decreasing concentrations of methionine (starting from 10 mM methionine and 5 mM cysteine, 2-fold dilutions were measured). Note: 10 mM cysteine led to partial growth inhibition (Supplementary Figure S11); this is why values starting from 5 mM are displayed. The gray and blue shaded boxes indicate the operational range of the promoter repressed methionine (blue) and repressed with cysteine (gray). (G) Performance of the protein degradation tags Ubi-M, Ubi-Y, and Ubi-R in *C. glabrata* using vector version 1.1. All experiments were run in biological triplicates (three transformants), and error bars represent the standard deviation. For all experiments, fluorescence measurements were taken after 16 h of growth (see Materials and Methods).

Next, we characterized all 19 YTK-derived constitutive promoters in *C. glabrata* using the highest expression vector 1.1 and one of the low expression vectors, vector version 2.2. All 19 YTK promoters were functional in *C. glabrata*, showing at least 3-fold expression over background in vector version 1.1 and covering 2 orders of magnitude in expression strength (Figure 1A). Expression was lower in vector version 2.2: 8- to 30-fold for the six strongest promoters (Figure 1B, Supplementary Figure S6). The exact order of promoter strength slightly varied between vectors, but a set of strong, medium, and weak promoters could be identified (Figure 1C). Several of the promoters showed different strength profiles in *C. glabrata* when compared to *S. cerevisiae* (Supplementary Figure S7) and the highest expression within the set of promoters was 2.7-fold lower in *C. glabrata* when compared to *S. cerevisiae* (Supplementary Figure S8B). To potentially reach higher expression in *C. glabrata* we tested two *C. glabrata*-derived central carbon metabolism promoters (Supplementary Figure S9). Neither showed higher expression than the YTK-promoters, but still medium to strong expression (27- to 49-fold over background). We tested a subset of seven promoters with a red fluorescent protein (mRuby2) as readout and obtained similar results (Supplementary Figure S10, Note S2).

We further tested four constitutive minimal synthetic promoters (MPs) originally engineered for *S. cerevisiae*,^35^ herein called MP1 to MP4 (Note S3). All four promoters were functional in *C. glabrata* yielding between 4- and 12-fold expression over background (Figure 1D). None of the MPs were strong, but they could be useful as short, low to medium expression promoters in *C. glabrata* and could likely be further optimized (Note S3).

### Three Inducible Promoters

As the YTK-derived galactose promoter is not functional in *C. glabrata*, we characterized two inducible *C. glabrata* promoters: the methionine and/or cysteine repressible promoter MET3p and the *C. glabrata* copper(II) sulfate (CuSO_4_)-inducible promoter MT-1p. In addition, we tested the YTK-derived CuSO_4_-inducible CUP1 promoter. The MET3 promoter in vector 1.1 allowed for a 17-fold change in Venus expression when using methionine as a repressor and promoter leakiness was 2-fold over background (Figure 1E and Supplementary Figure S12A). When using cysteine as a repressor, a 7-fold change in expression was achieved. The reduction in fold change was due to cysteine being a less effective repressor. At full cysteine repression (≥5 mM cysteine), leakiness was still 3-fold over background. However, using cysteine as a repressor widened the operational range of the promoter when compared to methionine (Figure 1E). Similar results, but lower expression levels were achieved when using vector version 2.2 (Figure 1F and Supplementary Figure S12B). Also, the two CuSO_4_-inducible promoters CUP 1p and MT-1p were functional in *C. glabrata* (Supplementary Figure S14, Note S4).

### Ubiquitin-Based Degradation Tags

N-terminal degradation tags (N-degrons) of different strength allow for tuning protein levels by protein turnover rates,^36^ and have proven to be useful tools for functional proteomics^37^ and genomics,^38^ fine-tuning of genetic circuit behavior,^39^ or temporal control over gene functions.^38^ Here we show that the ubiquitin-based YTK-derived degradation tags can be functionally ported into *C. glabrata* (Figure 1G, Supplementary Figure S15, and Note S5).

### A CRISPRi System for *C. glabrata*

Finally, we used the CgTK to build a CRISPRi system based on the deactivated Cas9 (dCas9) fused to the transcriptional repression domain Mxi1,^40^ in combination with the gRNA design featured in the YTK (Note S6). We chose to test repression of genes by a single gRNA as we are most interested in using the CRISPRi system for functional genomics studies where screens are based on the fact that repression by single gRNAs generates selectable phenotypes such that cells carrying a given gRNA can be enriched or depleted over growth and dilution cycles.

**Figure 2:**
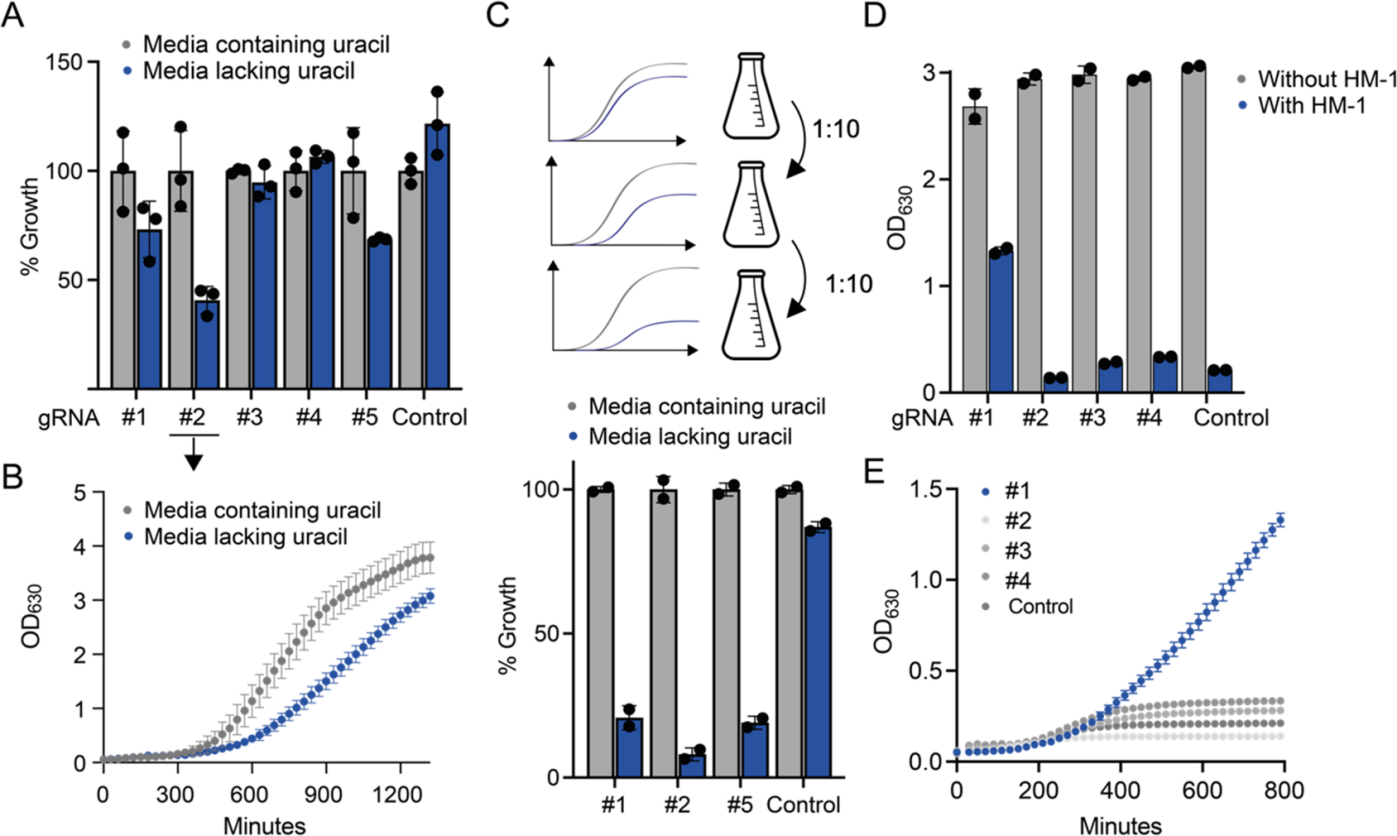
CRISPRi in *C. glabrata.* (A) Five gRNAs were tested for repression of the *URA3* gene by measuring growth in media lacking uracil. Percent growth (OD_630_) after 12 h when compared to the same strain grown in media with uracil is depicted. The control uses a gRNA that does not target the *URA3* promoter. (B) Full growth curve of the strain harboring the best performing gRNA #2 in media containing uracil and media lacking uracil. (C) Depletion of strains harboring gRNAs #1, #2, and #5 after three serial dilutions in media lacking uracil when compared to growth in media containing uracil. Strains were grown in the presence or absence of uracil in duplicate, and cultures were diluted 1:10 into fresh media after 7 to 10 h. Final OD_630_ after the third dilution round is given as percentage of growth in media containing uracil. (D) Four gRNAs were tested for repression of the *ALG3* gene by measuring growth in media containing the killer toxin HM-1. The control uses a gRNA that does not target the A*LG3* promoter. (E) Growth curves of the strains harboring the targeting or nontargeting RNAs in media containing HM-1.

We used the *URA3* gene (CAGL0I03080g) and the *ALG3* gene (CAGL0A04587g) to show the functionality of our CRISPRi system: The repression of *URA3* should lead to a selectable phenotype on media lacking uracil. *ALG3* is a functional yet unverified gene in *C. glabrata*, but its *S. cerevisiae* orthologue encodes for an alpha-1,3-mannosyltransferase involved in (membrane)-protein glycosylation. An *ALG3* deletion renders *S. cerevisiae* resistant to the yeast killer toxin HM-1. As we are interested in using the CRISPRi system to study resistant phenotypes to yeast killer toxins, we verified that an *ALG3* deletion would also render *C. glabrata* resistant to this toxin (Supplementary Figure S16) and subsequently aimed to verify that its repression by CRISPRi also changes the resistance profile. We designed four to five gRNAs targeting the promoters of *URA3* and *ALG3* in a window of −50 to +300 bp respective to the transcriptional start site (TSS) (Supplementary Table S5)^41^ based on previous design rules in *S. cerevisiae*(42,43) and mammalian cells. First, we tested the *URA3*p targeting CRISPRi system by measuring growth in media with and without uracil. For three out of the five gRNAs, we observed a slow growth phenotype in the absence of uracil (Figure 2A and B), which allowed to deplete the gRNA-encoding strains over three growth-and-dilution cycles to 4 to 10-fold (depending on gRNA) when compared to the same strain grown in the presence of uracil or a strain not expressing a targeting gRNA (Figure 2C). Next, we assessed the performance of the *ALG3*p targeting CRISPRi system: Here one out of the four tested gRNAs could be enriched in the presence of the killer toxin HM-1 (Figure 2D and E).

## Conclusion

Here we deliver a molecular cloning kit for the human fungal pathogen and biotechnology host *C. glabrata* by characterizing the performance of the YTK and by adding several new parts.

The strongest promoters generated expression levels 100-fold over background and allow a user to tune expression levels over 2 orders of magnitude. The herein characterized vector-set allows for further tuning expression strength as they have different copy number. To further enhance the expression strength of the promoters, it might be possible to choose better suited “seams” for the Golden Gate assembly of type 2 (promoter) and type 3 (open reading frame) parts as the YTK-inherent *Bgl*II site was shown to be suboptimal.^44^

The presented single-gRNA CRISPRi system is useful for library selections and could likely be extended to generate complete knock-down phenotypes (as required for metabolic engineering) by using several gRNAs that target the same promoter simultaneously. YTK-compatible multi-gRNA cloning strategies are readily available to extend the herein presented single-gRNA system.^45^

In summary, we think that the CgTK is a starting point for effective metabolic engineering and phenotypic characterization of *C. glabrata*.

## Supporting Information Available

Materials and Methods; Supplementary Tables S1–S5; Supplementary Figures S1–S17; Notes S1–S6 (PDF)

## References

[ref1] Kumar K.; Askari F.; Sahu M. S.; Kaur R. Candida glabrata: A Lot More Than Meets the Eye. Microorganisms 2019, 7 (2), 39 10.3390/microorganisms7020039. 30704135 PMC6407134

[ref2] Pfaller M. A.; Messer S. A.; Moet G. J.; Jones R. N.; Castanheira M. Candida Bloodstream Infections: Comparison of Species Distribution and Resistance to Echinocandin and Azole Antifungal Agents in Intensive Care Unit (ICU) and Non-ICU Settings in the SENTRY Antimicrobial Surveillance Program (2008–2009). Int. J. Antimicrob. Agents 2011, 38 (1), 65–69. 10.1016/j.ijantimicag.2011.02.016. 21514797

[ref3] Yapar N. Epidemiology and Risk Factors for Invasive Candidiasis. Ther. Clin. Risk Manag. 2014, 10 (1), 95–105. 10.2147/TCRM.S40160. 24611015 PMC3928396

[ref4] Chakrabarti A.; Sood P.; Rudramurthy S. M.; Chen S.; Kaur H.; Capoor M.; Chhina D.; Rao R.; Eshwara V. K.; Xess I.; Kindo A. J.; Umabala P.; Savio J.; Patel A.; Ray U.; Mohan S.; Iyer R.; Chander J.; Arora A.; Sardana R.; Roy I.; Appalaraju B.; Sharma A.; Shetty A.; Khanna N.; Marak R.; Biswas S.; Das S.; Harish B. N.; Joshi S.; Mendiratta D. Incidence, Characteristics and Outcome of ICU-Acquired Candidemia in India. Intensive Care Med. 2015, 41 (2), 285–295. 10.1007/s00134-014-3603-2. 25510301

[ref5] Diekema D.; Arbefeville S.; Boyken L.; Kroeger J.; Pfaller M. The Changing Epidemiology of Healthcare-Associated Candidemia over Three Decades. Diagn. Microbiol. Infect. Dis. 2012, 73 (1), 45–48. 10.1016/j.diagmicrobio.2012.02.001. 22578938

[ref6] Fisher M. C.; Hawkins N. J.; Sanglard D.; Gurr S. J. Worldwide Emergence of Resistance to Antifungal Drugs Challenges Human Health and Food Security. Science (80-.). 2018, 360 (6390), 739–742. 10.1126/science.aap7999. 29773744

[ref7] Healey K. R.; Zhao Y.; Perez W. B.; Lockhart S. R.; Sobel J. D.; Farmakiotis D.; Kontoyiannis D. P.; Sanglard D.; Taj-Aldeen S. J.; Alexander B. D.; Jimenez-Ortigosa C.; Shor E.; Perlin D. S. Prevalent Mutator Genotype Identified in Fungal Pathogen Candida Glabrata Promotes Multi-Drug Resistance. Nat. Commun. 2016, 7 (1), 1–10. 10.1038/ncomms11128. PMC5603725 27020939

[ref8] Whaley S. G.; Berkow E. L.; Rybak J. M.; Nishimoto A. T.; Barker K. S.; Rogers P. D. Azole Antifungal Resistance in Candida Albicans and Emerging Non-Albicans Candida Species. Front. Microbiol. 2017, 7, 2173 10.3389/fmicb.2016.02173. 28127295 PMC5226953

[ref9] Whaley S. G.; Rogers P. D. Azole Resistance in Candida Glabrata. Curr. Infect. Dis. Rep. 2016, 18 (12), 41 10.1007/s11908-016-0554-5. 27761779

[ref10] Healey K. R.; Nagasaki Y.; Zimmerman M.; Kordalewska M.; Park S.; Zhao Y.; Perlin D. S. The Gastrointestinal Tract Is a Major Source of Echinocandin Drug Resistance in a Murine Model of Candida Glabrata Colonization and Systemic Dissemination. Antimicrob. Agents Chemother. 2017, 10.1128/AAC.01412-17. PMC5700336 28971865

[ref11] Van Ende M.; Timmermans B.; Vanreppelen G.; Siscar-Lewin S.; Fischer D.; Wijnants S.; Romero C. L.; Yazdani S.; Rogiers O.; Demuyser L.; Van Zeebroeck G.; Cen Y.; Kuchler K.; Brunke S.; Van Dijck P. The Involvement of the Candida Glabrata Trehalase Enzymes in Stress Resistance and Gut Colonization. Virulence 2021, 12 (1), 329–345. 10.1080/21505594.2020.1868825. 33356857 PMC7808424

[ref12] Roetzer A.; Gabaldón T.; Schüller C. From Saccharomyces Cerevisiae to Candida Glabrata in a Few Easy Steps: Important Adaptations for an Opportunistic Pathogen. Fems Microbiol. Lett. 2011, 314 (1), 1 10.1111/j.1574-6968.2010.02102.x. 20846362 PMC3015064

[ref13] Timmermans B.; De Las Peñas A.; Castaño I.; Van Dijck P. Adhesins in Candida Glabrata. J. Fungi 2018, 4 (2), 60 10.3390/jof4020060. PMC6023314 29783771

[ref14] Hassan Y.; Chew S. Y.; Than L. T. L. Candida Glabrata: Pathogenicity and Resistance Mechanisms for Adaptation and Survival. J. Fungi 2021, 7 (8), 667 10.3390/jof7080667. PMC8398317 34436206

[ref15] Xu N.; Ye C.; Chen X.; Liu J.; Liu L.; Chen J. Genome Sequencing of the Pyruvate-Producing Strain Candida Glabrata CCTCC M202019 and Genomic Comparison with Strain CBS138. Sci. Rep. 2016, 6 (1), 1–10. 10.1038/srep34893. 27713500 PMC5054605

[ref16] Luo Z.; Zeng W.; Du G.; Chen J.; Zhou J. Enhanced Pyruvate Production in Candida Glabrata by Engineering ATP Futile Cycle System. ACS Synth. Biol. 2019, 8 (4), 787–795. 10.1021/acssynbio.8b00479. 30856339

[ref17] Zhang D.; Liang N.; Shi Z.; Liu L.; Chen J.; Du G. Enhancement of α-Ketoglutarate Production in Torulopsis Glabrata: Redistribution of Carbon Flux from Pyruvate to α-Ketoglutarate. Biotechnol. Bioprocess Eng. 2009, 14, 134–139. 10.1007/s12257-008-0169-2.

[ref18] Chen X.; Zhu P.; Liu L. Modular Optimization of Multi-Gene Pathways for Fumarate Production. Metab. Eng. 2016, 33, 76–85. 10.1016/j.ymben.2015.07.007. 26241189

[ref19] Li S.; Xu N.; Liu L.; Chen J. Engineering of Carboligase Activity Reaction in Candida Glabrata for Acetoin Production. Metab. Eng. 2014, 22, 32–39. 10.1016/j.ymben.2013.12.005. 24365210

[ref20] Chen X.; Xu G.; Xu N.; Zou W.; Zhu P.; Liu L.; Chen J. Metabolic Engineering of Torulopsis Glabrata for Malate Production. Metab. Eng. 2013, 19, 10–16. 10.1016/j.ymben.2013.05.002. 23707987

[ref21] Gao X.; Xu N.; Li S.; Liu L. Metabolic Engineering of Candida Glabrata for Diacetyl Production. PLoS One 2014, 9, e89854 10.1371/journal.pone.0089854. 24614328 PMC3948628

[ref22] Budin I.; Keasling J. D. Synthetic Biology for Fundamental Biochemical Discovery. Biochemistry 2019, 58 (11), 1464–1469. 10.1021/acs.biochem.8b00915. 30350947

[ref23] Zordan R. E; Ren Y.; Pan S.-J.; Rotondo G.; Penas A. D. L.; Iluore J.; Cormack B. P Expression Plasmids for Use in Candida Glabrata. G3 Genes, Genomes, Genet. 2013, 3 (9), 1675–1686. 10.1534/g3.113.006908. PMC3789792 23934995

[ref24] Enkler L.; Richer D.; Marchand A. L.; Ferrandon D.; Jossinet F. Genome Engineering in the Yeast Pathogen Candida Glabrata Using the CRISPR-Cas9 System. Sci. Rep. 2016, 6 (1), 35766 10.1038/srep35766. 27767081 PMC5073330

[ref25] Maroc L.; Fairhead C. A New Inducible CRISPR-Cas9 System Useful for Genome Editing and Study of Double-Strand Break Repair in Candida Glabrata. Yeast 2019, 36 (12), 723–731. 10.1002/yea.3440. 31423617

[ref26] Castaño I.; Kaur R.; Pan S.; Cregg R.; De Las Peñas A.; Guo N.; Biery M. C.; Craig N. L.; Cormack B. P. Tn7-Based Genome-Wide Random Insertional Mutagenesis of Candida Glabrata. Genome Res. 2003, 13 (5), 905–915. 10.1101/gr.848203. 12695329 PMC430877

[ref27] Gale A. N.; Sakhawala R. M.; Levitan A.; Sharan R.; Berman J.; Timp W.; Cunningham K. W. Identification of Essential Genes and Fluconazole Susceptibility Genes in *Candida Glabrata* by Profiling *Hermes* Transposon Insertions. G3 (Bethesda) 2020, 10 (10), 3859–3870. 10.1534/g3.120.401595. 32819971 PMC7534453

[ref28] Schwarzmüller T.; Ma B.; Hiller E.; Istel F.; Tscherner M.; Brunke S.; Ames L.; Firon A.; Green B.; Cabral V.; Marcet-Houben M.; Jacobsen I. D.; Quintin J.; Seider K.; Frohner I.; Glaser W.; Jungwirth H.; Bachellier-Bassi S.; Chauvel M.; Zeidler U.; Ferrandon D.; Gabaldón T.; Hube B.; d’Enfert C.; Rupp S.; Cormack B.; Haynes K.; Kuchler K. Systematic Phenotyping of a Large-Scale Candida Glabrata Deletion Collection Reveals Novel Antifungal Tolerance Genes. PLoS Pathog. 2014, 10 (6), e1004211 10.1371/journal.ppat.1004211. 24945925 PMC4063973

[ref29] Engler C.; Kandzia R.; Marillonnet S. A One Pot, One Step, Precision Cloning Method with High Throughput Capability. PLoS One 2008, 3 (11), e3647 10.1371/journal.pone.0003647. 18985154 PMC2574415

[ref30] Lee M. E.; DeLoache W. C.; Cervantes B.; Dueber J. E. A Highly Characterized Yeast Toolkit for Modular, Multipart Assembly. ACS Synth. Biol. 2015, 4 (9), 975–986. 10.1021/sb500366v. 25871405

[ref31] Chen B.; Lee H. L.; Heng Y. C.; Chua N.; Teo W. S.; Choi W. J.; Leong S. S. J.; Foo J. L.; Chang M. W. Synthetic Biology Toolkits and Applications in Saccharomyces Cerevisiae. Biotechnol. Adv. 2018, 36 (7), 1870–1881. 10.1016/j.biotechadv.2018.07.005. 30031049

[ref32] Moore S. J.; Lai H.-E.; Kelwick R. J. R.; Chee S. M.; Bell D. J.; Polizzi K. M.; Freemont P. S. EcoFlex: A Multifunctional MoClo Kit for *E. Coli* Synthetic Biology. ACS Synth. Biol. 2016, 5 (10), 1059–1069. 10.1021/acssynbio.6b00031. 27096716

[ref33] Chiasson D.; Giménez-Oya V.; Bircheneder M.; Bachmaier S.; Studtrucker T.; Ryan J.; Sollweck K.; Leonhardt H.; Boshart M.; Dietrich P.; Parniske M. A Unified Multi-Kingdom Golden Gate Cloning Platform. Sci. Rep. 2019, 9 (1), 1–12. 10.1038/s41598-019-46171-2. 31300661 PMC6626145

[ref34] Karim A. S.; Curran K. A.; Alper H. S. Characterization of Plasmid Burden and Copy Number in Saccharomyces Cerevisiae for Optimization of Metabolic Engineering Applications. FEMS Yeast Res. 2013, 13 (1), 107–116. 10.1111/1567-1364.12016. 23107142 PMC3546148

[ref35] Redden H.; Alper H. S. The Development and Characterization of Synthetic Minimal Yeast Promoters. Nat. Commun. 2015, 6 (1), 1–9. 10.1038/ncomms8810. PMC4518256 26183606

[ref36] Hackett E. A.; Esch R. K.; Maleri S.; Errede B. A Family of Destabilized Cyan Fluorescent Proteins as Transcriptional Reporters in *S*. Cerevisiae. Yeast 2006, 23 (5), 333–349. 10.1002/yea.1358. 16598699

[ref37] Ross A. B.; Langer J. D.; Jovanovic M. Proteome Turnover in the Spotlight: Approaches, Applications, and Perspectives. Mol. Cell. Proteomics 2021, 20, 100016 10.1074/mcp.R120.002190. 33556866 PMC7950106

[ref38] Trauth J.; Scheffer J.; Hasenjäger S.; Taxis C. Synthetic Control of Protein Degradation during Cell Proliferation and Developmental Processes. ACS Omega 2019, 4 (2), 2766–2778. 10.1021/acsomega.8b03011.

[ref39] Chassin H.; Müller M.; Tigges M.; Scheller L.; Lang M.; Fussenegger M. A Modular Degron Library for Synthetic Circuits in Mammalian Cells. Nat. Commun. 2019, 10.1038/s41467-019-09974-5. PMC6494899 31043592

[ref40] Xu D.; Jiang B.; Ketela T.; Lemieux S.; Veillette K.; Martel N.; Davison J.; Sillaots S.; Trosok S.; Bachewich C.; Bussey H.; Youngman P.; Roemer T. Genome-Wide Fitness Test and Mechanism-of-Action Studies of Inhibitory Compounds in Candida Albicans. PLoS Pathog. 2007, 3 (6), e92 10.1371/journal.ppat.0030092. 17604452 PMC1904411

[ref41] Aoyama T.; Nakayama H.; Ueno K.; Inukai T.; Tanabe K.; Nagi M.; Bard M.; Chibana H. Genome-Wide Survey of Transcriptional Initiation in the Pathogenic Fungus. Candida Glabrata. Genes to Cells 2014, 19 (6), 478–503. 10.1111/gtc.12147. 24725256

[ref42] McGlincy N. J.; Meacham Z. A.; Reynaud K. K.; Muller R.; Baum R.; Ingolia N. T. A Genome-Scale CRISPR Interference Guide Library Enables Comprehensive Phenotypic Profiling in Yeast. BMC Genomics 2021, 22 (1), 1–17. 10.1186/s12864-021-07518-0. 33757429 PMC7986282

[ref43] Smith J. D.; Suresh S.; Schlecht U.; Wu M.; Wagih O.; Peltz G.; Davis R. W.; Steinmetz L. M.; Parts L.; St.Onge R. P. Quantitative CRISPR Interference Screens in Yeast Identify Chemical-Genetic Interactions and New Rules for Guide RNA Design. Genome Biol. 2016, 10.1186/s13059-016-0900-9. PMC4784398 26956608

[ref44] Otto M.; Skrekas C.; Gossing M.; Gustafsson J.; Siewers V.; David F. Expansion of the Yeast Modular Cloning Toolkit for CRISPR-Based Applications, Genomic Integrations and Combinatorial Libraries. ACS Synth. Biol. 2021, 10 (12), 3461–3474. 10.1021/acssynbio.1c00408. 34860007 PMC8689691

[ref45] McCarty N. S.; Shaw W. M.; Ellis T.; Ledesma-Amaro R. Rapid Assembly of GRNA Arrays via Modular Cloning in Yeast. ACS Synth. Biol. 2019, 8 (4), 906–910. 10.1021/acssynbio.9b00041. 30939239

