# Supplementary material for: A Modular Cloning Toolkit Including CRISPRi for the Engineering of the Human Fungal Pathogen and Biotechnology Host Candida glabrata
Source: ACS Synth Biol. 2023 Apr 12;12(4):1358–63. doi: 10.1021/acssynbio.2c00560 (PMC10127446; doi:10.1021/acssynbio.2c00560)
Supplement: Supplementary file 1 — sb2c00560_si_001.pdf [file sb2c00560_si_001.pdf]

Supplementary material

A modular cloning toolkit including CRISPRi for the engineering of the human fungal pathogen and biotechnology host *Candida glabrata*

Sonja Billerbeck[1]*, Rianne C Prins[1], Malte Marquardt[1]

[1] Department for Molecular Microbiology, Groningen Biomolecular Sciences and Biotechnology Institute, University of Groningen, Nijenborgh 7, 9747 AG Groningen, The Netherlands

*correspondence: s.k.billerbeck@rug.nl

CONTENT

1. Materials and Methods
Materials
Strains
Plasmids, primers and synthetic DNA
Media and inductions
Yeast transformation
Cloning of new parts into the entry vectors (pYTK01) and Golden Gate assembly
Fluorescence measurements and fluorescence calibration
Growth measurement
Plasmid/DNA extraction and qPCR

2. Supplementary Tables
Supplementary Table 1. YTK parts that were used for vector assembly or re-characterized in *C. glabrata.*
Supplementary Table 2. New parts added to the *Cg*TK.
Supplementary Table 3. Sequences of new *Cg*TK parts.
Supplementary Table 4. Primers used in this study.
Supplementary Table 5. gRNA sequences and targeted promoters.

3. Supplementary Figures
Supplementary Figure 1. Overview on the CgTK
Supplementary Figure 2. Growth of *C. glabrata* transformed with empty vectors.
Supplementary Figure 3. Growth of *C. glabrata* transformed with Venus-expression vectors.
Supplementary Figure 4. Differences in expression level of Venus from the various vectors.
Supplementary Figure 5. qPCR analysis of relative cellular abundance of various vectors.
Supplementary Figure 6. Fold-difference in expression across various promoters when comparing the vector versions 1.1 and version 2.2.
Supplementary Figure 7. Expression levels of YTK promoters and minimal promoters in *S. cerevisiae.*
Supplementary Figure 8. Comparison of promoter performance in *C. glabrata* and *S. cerevisiae*.
Supplementary Figure 9. Comparison of *C. glabrata* derived TDH3 and PGK1 promoters with the strongest YTK-derived promoters.
Supplementary Figure 10. Performance of seven YTK promoters in *C. glabrata and S. cerevisiae* using red fluorescence (mRuby2) as readout.
Supplementary Figure 11. Growth of *C. glabrata* in the presence of methionine and cysteine.
Supplementary Figure 12. ON and OFF state of Met3 promoter.
Supplementary Figure 13. Growth of *C. glabrata* and *S. cerevisiae* in the presence of copper (II) sulfate.
Supplementary Figure 14. Performance of the MT-I and the CUP1 promoters.
Supplementary Figure 15. Performance of the protein degradation tags.
Supplementary Figure 16. Resistance of *C. glabrata* DeltaRHK1/ALG3 to HM-1.
Supplementary Figure 17. Basal expression from the three copper inducible promoters when cloned into vector version 1.1 or 2.2 in *C. glabrata* and *S. cerevisiae*.

4. Notes
Note 1: Growth burden caused by ScCEN/ARS Venus expression vectors.
Note 2: Performance of seven YTK promoters when driving the red fluorescent protein mRuby.
Note 3: Design of minimal promoters and potential for optimization.
Note 4. Performance of $CuSO_4$-inducible promoters in *C. glabrata* and *S. cerevisiae*.
Note 5: Degradation tags.
Note 6: Outline of the CRISPRi system.

1. MATERIALS AND METHODS

Materials

Phire Green Hot Start II PCR Master Mix was used for all PCR reactions and was purchased from Thermo Fisher (#F126L). Restriction enzymes BsaI-HF®v2 (#R3733S) and BsmBI-v2 (#R0739S) and T7 ligase (#M0318S) were used for the Golden Gate reactions and were obtained from New England Biolabs (NEB). Media components were obtained from BD Bioscience and Sigma-Aldrich. Primers and synthetic DNA (gBlocks) were obtained from Integrated DNA Technologies (IDT); Primers used in this study are listed in **Supplementary Table 4**. Plasmids were cloned and amplified in *E. coli* DH5α. Clear, round bottom 96-well microtiter plates (Costar) were used for culturing. Black, clear-bottom 96-well microtiter plates (Costar) were used for fluorescence measurements. Fluorescence and optical density measurements were performed in a SynergyMx (Biotek) plate reader at 630 nm (optical density), 561ex/610em (red fluorescence) and 488ex/530em (green fluorescence).

Strains

Experiments were either performed in *C. glabrata* ATCC 2001 HTL$^-$ or its derivative HTLU$^-$ and in *S. cerevisiae* BY4741 (*MATa leu2Δ0 met15Δ0 ura3Δ0 his3Δ1*).[1] *E. coli* DH5α was used for cloning.
Strain *C. glabrata* ATCC 2001 HTL$^-$ DeltaRHK1/ALG3[2] (ORF CAGL0A04587g deleted) was used as a control for the ALG3 CRISPRi experiments.

Plasmids, primers and synthetic DNA

Plasmids are listed in **Supplementary Table 1 and 2,** sequences of primers are listed in **Supplementary Table 4,** synthetic DNA (gBlocks, IDT) are listed in **Supplementary Table 3.**

Media and inductions

Yeast strains were routinely grown in synthetic drop-out media (SD) with 2% (w/v) Dextrose (Fisher Scientific), 0.67% (w/v) Yeast Nitrogen Base without amino acids (VWR International), and a self-made 20-fold amino acid drop-out mix.[3] Nourseothricin was used at 100 µg/mL final concentration in liquid or solid YPD media (yeast extract 2% , peptone 1%, dextrose 2%).

E. coli was grown in Luria Broth (LB) media. To select for *E. coli* plasmids with drug-resistant genes, ampicillin (Sigma-Aldrich) or kanamycin (Sigma-Aldrich) were used at final concentrations of 75-200 µg/mL and 50 µg/mL, respectively. Agar was added to 2% for preparing solid yeast and bacterial media. Copper inductions were performed in SD media with 0-2 mM copper (II) sulfate added. Methionine/cysteine repressions were performed in SD media lacking methionine in the drop-out mix, with 0-10 mM methionine or cysteine added.

Yeast transformation

C. glabrata and *S. cerevisiae* cells were transformed with plasmid DNA using the lithium acetate transformation protocol described before.[4]

Cloning of new parts into the entry vectors (pYTK01) and Golden Gate assembly

Primers with overhangs for BsmBI-based cloning into the entry level vector pYTK01 were designed as instructed in Lee *at al.*[5] Golden Gate reactions were performed as instructed in Lee *at al.*[5] In brief: 0.5 µL of each DNA insert or plasmid, 1 µL T4 DNA Ligase buffer (NEB), 0.5 µL T7 DNA Ligase (NEB), 0.5 µL restriction enzyme, and water to bring the final volume to 10 µL. The restriction enzymes used were either BsaI or BsmBI (both 10 000 U/mL from NEB).

Reaction mixtures were incubated in a thermocycler according to the following program: 25 cycles of digestion and ligation (42 °C for 2 min, 16 °C for 5 min) followed by a final digestion step (60 °C for 10 min), and a heat inactivation step (80 °C for 10 min). In some cases, where noted in the text, the final digestion and heat inactivation steps were omitted.

On a technical note; Golden Gate-based cloning of dCas9-MxiI was less reliable and at least 5 white colonies needed to be picked after green white screening and their plasmids control digested in order to identify one correct assembly. This is in contrast to the usually highly efficient YTK-based Golden Gate assembly were almost 100% of white colonies carry correct assemblies.

Fluorescence measurements and fluorescence calibration

Three colonies were picked and grown in 200 µL of media in 96-well plates (clear, round bottom) at 30°C in an orbital shaker (microtitre plate shaker incubator SI505 from Stuart) shaking at 950 rpm until saturated. Cultures were diluted 1:100 in fresh media, grown for 16 h at the same conditions as the overnight cultures, and then diluted 1:2 in water in 96-well black clear-bottom plates, and fluorescence was measured in a SynergyMx plate reader. For the copper inductions, saturated cultures were diluted 1:100 in fresh media with different concentrations of copper (II) sulfate and grown for 16 h. For the methionine/cysteine repression, saturated cultures (grown in the presence of methionine, OFF state) were washed 5 times in 5 volumes of water and diluted 1:100 in fresh media with different concentrations of methionine or cysteine added and grown for 16 h.

Excitation and emission wavelengths used to measure fluorescent proteins were 488 nm/530 nm for Venus and 561 nm/610 nm for mRuby2. Raw fluorescence values were first corrected by subtracting the average autofluorescence of a black clear-bottom plate, then normalized to the OD_{630} of the cultures, and then normalized to the respective calibrant dye (fluorescein for Venus and sulforhodamine-101 for mRuby2). Calibrating arbitrary fluorescence units into nM calibrant dye fluorescence was performed as described before.[6] Linear regression was used to derive the conversion factor.

Growth measurements

Growth curves were recorded in sterile, transparent round-bottom 96-well plates using 200 µL total culture volume, cultured at 30°C in a SynergyMx plate reader (high orbital shaking). Cells were seeded at an OD_{630} of approximately 0.03 and culture turbidity (OD_{630}) was recorded every 30 minutes for 20 to 24 h. For gRNA depletion experiments, cells were grown in 50 mL shake flasks and since the later optical density values were outside the linear range of the photodetector, all optical density values were first corrected using the following formula to calculate true optical density values:

$$OD_{true} = k \cdot OD_{meas} / (OD_{sat} - OD_{meas}) \quad \text{(Eq. 1)}$$

where OD_{meas} is the measured optical density, OD_{sat} is the saturation value of the photodetector (2.568 for our instrument, as experimentally determined) and k is the true optical density at which the detector reaches half saturation of the measured optical density (2.075 for our instrument, as experimentally determined). For gRNA depletion experiments shown in **Figure 2C**, cells were grown in 50 mL shake flasks and diluted 1:10 every 7 to 10 h. Optical density was measured in 1 mL cuvettes in a spectrophotometer (Nanospec, Amersham Bioscience). For selections in the presence of HM-1, HM-1 was concentrated from an in-house production strain and used as 0.4x diluted supernatant in the final assay.

Plasmid/DNA extraction and qPCR

Two different protocols for extracting DNA or plasmids from *C. glabrata* were employed:

Plasmid extraction: 4 mL of media were inoculated with a *C. glabrata* colony harboring the targeted plasmids and incubated at 30 °C shaking at 200 rpm for 16 – 18 h. The equivalent of 5 OD units were used for extraction. The cells were centrifuged at 10,000 g for 15 sec and resuspended in 1 M Sorbitol, 100 mM EDTA, 14 mM β-mercaptoethanol, 20 U/mL lyticase (Sigma #L2524-10KU) for 1 h. For further processing the GeneJET Plasmid Miniprep Kit (Thermo Fisher) was utilized as instructed. Plasmid DNA was eluded in 30 µL of water.

Total DNA extraction from C. glabrata: For total DNA extraction 4 mL of media were inoculated with a *C. glabrata* colony harboring the targeted plasmids and incubated at 30 °C shaking at 200 rpm for 16 – 18 h. The equivalent of 10 OD Units used for DNA extraction. For this the cells were pelleted and washed in 50 mM EDTA and subsequently incubated in 50 mM EDTA, 20 U/mL lyticase (Sigma #L2524-10KU) at 30°C for 1 h. The pellet was resuspended in 50 mM EDTA, 2% SDS and incubated at 65 °C for 15 min before adding 1:1 potassium acetate. After centrifugation at 20,000 *g* for 10 minutes the supernatant was transferred to a fresh tube and isopropanol was added 1:1. The solution was again centrifuged at 20,000 *g* for 10 min, after which the DNA pellet was washed with 70% EtOH, air-dried and resuspended in 25 µL of water.

qPCR: qPCR reactions containing 4 µL sample, 12.5 µL 2x SensiFAST SYBR Hi-ROX Mix (Meridian Bioscience), 1 µL forward and reverse Primer (10 mM) (see **Supplementary Table 4** for primer sequences) and water for a total volume of 25 µL was performed in white 96-well plates. Relative copy numbers and relative normalized copy numbers were determined in three biological replicates, which were measured in three technical replicates respectively. Standard cycling conditions as recommended by the manufacturer of the reaction mix were employed for data generation. For determining relative normalized expression of plasmid per cell, total DNA extract sample was added to the reaction mixture. The amount of measured plasmid (primers Venus-fw and Venus-rev) was normalized for the amount of actin (Actin-fw and Actin-rv) in each sample and the average of each technical replicate group was calculated and again averaged within the biological group resulting in an average ΔCq value per biological replicate group. The ΔCq values of each biological replicate group were then cross-compared yielding ΔΔCq values. The fold-changes corresponding to these ΔΔCq values are shown in **Supplementary Figure 5**. For relative copy numbers samples of plasmid extraction were added to the reaction mixture. The amount of plasmid per sample was measured (primers Venus-fw and Venus-rev) and the relative amount determined without normalizing for actin by directly averaging the technical and thereafter the biological replicates. The fold-changes corresponding to the ΔCq values of the cross-comparison between the biological replicate groups **Supplementary Figure 5**.

2. SUPPLEMENTARY TABLES

YTK#	Name	Type
YTK parts re-used for vector backbones		
pYTK08	ConLS’	1
pYTK73	ConRE’	5
pYTK74	URA3	6
pYTK75	LEU2	6
pYTK76	HIS3	6
pYTK78	NourseothricinR	6
pYTK84	KanR-ColE1	8
pYTK81	CEN6/ARS4	7
pYTK47	GFP dropout	2/3/4
YTK promoters characterized in *C. glabrata*		
pYTK09	pTDH3	2
pYTK10	pCCW12	2
pYTK11	pPGK1	2
pYTK12	pHHF2	2
pYTK13	pTEF1	2
pYTK14	pTEF2	2
pYTK15	pHHF1	2
pYTK16	pHTB2	2
pYTK17	pRPL18B	2
pYTK18	pALD6	2
pYTK19	pPAB1	2
pYTK20	pRET2	2
pYTK21	pRNR1	2
pYTK22	pSAC6	2
pYTK23	pRNR2	2
pYTK24	pPOP6	2
pYTK25	pRAD27	2
pYTK26	pPSP2	2
pYTK27	pREV1	2
pYTK31	pCUP1	2
YTK fluorescent proteins characterized in *C. glabrata*		
pYTK33	Venus	3
pYTK34	mRuby2	3
YTK terminators characterized in *C. glabrata*		
pYTK51	tENO1	4
YTK degrons characterized in *C. glabrata*		
pYTK41	Ubi-M	3a
pYTK42	Ubi-Y	3a
pYTK43	Ubi-R	3a

Supplementary Table 1. YTK parts that were used for vector assembly or re-characterized in *C. glabrata.*

Supplementary Table 2. New parts added to the *Cg*TK.

CgTK#	Name	Part type	Source of DNA and reference
**Level 0 parts**			
CgTK parts for creating vector backbones			
pCgTK01	*Cg*CEN/ARS	7	pCU-Met3-GFP (Addgene# 45337)[7]
pCgTK02	CgTRP1	6	Genomic DNA of ATCC2001, BsaI/BsmBI sites removed
CgTK promoter parts			
pCgTK03	CgMET3p	2	pCU-Met3-GFP (Addgene# 45337)[7]
pCgTK04	CgMT1p	2	Genomic DNA of ATCC2001
pCgTK05	CgTDH3p	2	Genomic DNA of ATCC2001
pCgTK06	CgPGK1p	2	Genomic DNA of ATCC2001
pCgTK07	MP1 (Core 1, UAS c-e-f)	2	Ordered as synthetic DNA, design derived from Redden *et al.*[8]
pCgTK08	MP2 (Core 5, UAS c-e-f)	2	
pCgTK09	MP3 (Core 8, UAS c-e-f)	2	
pCgTK10	MP4 (Core 9, UAS c-e-f)	2	
CgTK *CRISPRi* parts			
pCgTK11	NLS-dCas9-Mxi1 (BsaI- and BsmBI-site free)	3	Ordered as synthetic DNA, design based on Smith *at al.*[9] The original construct contained 3 BsaI sites and 1 BsmBI site. We modified these 4 regions to remove all BsaI and BsmBI sites.
**Preassembled vectors**			
CgTK vectors for cloning (multiple) TUs			
CgTK13	Version 1.1 (ScCEN6/ARS4, *HIS3*)		
CgTK14	Version 1.2 (ScCEN6/ARS4, *TRP1*)		
CgTK15	Version 1.3 (ScCEN6/ARS4, *LEU2*)		
CgTK16	Version 1.4 (ScCEN6/ARS4, *URA3*)		
CgTK17	Version 1.5 (ScCEN6/ARS4, *NAT1*)		
CgTK18	Version 2.1 (CgCEN/ARS, *HIS3*)		
CgTK19	Version 2.2 (CgCEN/ARS, *TRP1*)		
CgTK20	Version 2.3 (CgCEN/ARS, *LEU2*)		
CgTK21	Version 2.4 (CgCEN/ARS, *URA3*)		
CRISPRi vectors			
CgTK22	Vector 2.2_HHF1p_dCas9-Mxi1_ENO1t		

Supplementary Table 3. Sequences of new *Cg*TK parts.
The part-specific overhangs are indicated in bold. The sequences for the minimal promoter units are annotated as follows: Capitals, green: upstream activating sequence c; Capitals, dark blue: upstream activating sequence e; Capitals, red: upstream activating sequence f; Bold, black: minimal core promoter; Blue: spacer.

CgTK number	Description	Sequence
pCgTK01	*Cg*CEN/ARS	**GAGT**ttgcagtcgtactggatctgtgaatctattagtatatatgaattaaagtagcttgtacatattatt<br>ctgttgaatcatatcgcagagcattggttgaaatccaaaatataaaaatgtaatatcacaaaaaataata<br>ctaattctaacattaatgggtcagatttttagtgaatacttaaatttataatatcgtctatttaagctagcaaa<br>tggaacaacatttaaagtaagaacatcatatctacatgaaaatgtatatttcaatctgactaataacgca<br>gagcacatctttcagtgatgtctgtcacatgatcaaaaagaattgtatttaaataattcataataaaagctt<br>aaaaaattacaataatgaaaataaagtaaataatgacatgggtaagagttccgaaatagaatcttagtg<br>tacaaaacaaaaattgcatcattagagatcccca**CCGA**
pCgTK02	Cg*TRP1*p-*TRP1*-*TRP1*t	**TACA**agtgcaaaggcatttcctttgccacacacactaccataactagtctcggtattaactggctca<br>acaaacgataagagccaaagaggaagaaaaaaaaggtatttaaaggatgtatttctttatctcctgatt<br>agatttcttacttgtacagaaactgtaactaaaaaaaaacaacaacaaaacacaacacatacataatgg<br>ctagaaccaagcaaaccgcaagaaagtctactggtggtaaggccccaagaaagcaactagcttcta<br>aggctgccagaaaatccgctccatctaccggtggtgtcaagaagcctcacagatataagccaggtac<br>cgtcgctttgagagaaatcagaagattccaaaagtctactgaacttttgatcagaaagttgcctttccaa<br>agactagtcagagaaatcgcccaagatttcaagaccgatctaagattccaatcctctgccatcggtgc<br>tctacaggaatccgttgaagcttacttagtctctttgttcgaagacaccaacttggccgctatccacgct<br>aagcgtgttaccatccaaaagaaggatatcaagttggctagaagattgagaggtgaaagatcctaag<br>cacacagggattgttgtacaattactttttatgattttagatacacacaaatcttttctacgattacctttcatt<br>tattttcgtttcatgttttattaatcattctttttgtatatttcacatcgggtggggtttttatattaaattttaataa<br>cataatcttttaaagtttctactttctatcctgtatattatttccatatctatatctatattttcaagcttgttcaca<br>tccttatcggaattgttccagcgtttttgtacagaaaatgatgttatggtaacgagctatatcagacctact<br>agtcttatagagctgtacagagtcttggaaacataaccaacacaccaacaagatgtcatttgattcgtta<br>ctcgacaagaatgataagctggtaaaagtttgcgggattcaaaccgtcgaggctgccgaaactgcgc<br>ttcaagcaggcgctgatttgatagggatcatatgtgtccccaacaggaagcggactatcgagagcgc<br>tgtggctcgtgaaatatccaaattgattcacaaatcaggtactacaaagctggtgggggtgttcagga<br>atcaatctgttgaggacgtacatcggctttctgaggaatatgaccttgacataatccaattacatggtga<br>tgaatcatggccagagtactataacgtcattaagaaaccaataatcaaaagagtcatattccctagaga<br>tgtcgatgttgtaacacaagtgtgtcaaagaaaacccttggtatgtctaccattgttcgactccgaggca<br>ggcggtacaggtgaaaagctggactggtccagtatttcctcctgggcaagcgaacagaataacgtat<br>tttatatactagcgggtggactaactgcagaaaacgtcatggaggcggtggggttgcctggtgtaatt<br>ggtgttgacgtatcaggtggtgtagagactgacggcgtcaaagacaatgacaagataattaagtatgt<br>acaaaatgcaaagaaacaatgaattacgaagagaagtctcaatgaggttttacaaacaatcctataat<br>acatatatgccactatgatatactttgaatactgagtatactgactcattatattctttaattgtagtttctcaa<br>aaatattcatacaggctagctatgaattcatgctataatttccattttgttcatgtaagatacacatággga<br>taaattgtgataactaatcagccgacttccgggtaccgttaatattatgcgtacttgttttcatgcgttgcg<br>ttattcaccctctttttaaatcgtcagtcgaattcgtaaacttcttaaataatatcgatattgattacatctgttt<br>agtaatcaaaacattcgataagtcagaattcattcaatgcataaaaatctgtgaatactgaggaacaatt<br>ggagtttcagtaaaaagtcttgcaaagcatgccaagttcagtgactgtcttacacatattaccacagaa<br>gtatcttcaccaaatttagtc**GAGT**
pCgTK03	CgMET3p	**AACG**ggcttatatgtatggaggtagggaactgcagtttattgttctgttaagctcccagtaatgcaa<br>gacggctaaatcatatgactgccactttgttgatcctgaagaaaatgacaacaagtagaaagtgatata<br>atacacgtggtatccgtagtatggtatcaggagatagccacatcacatgatctctaaaaccccccgtc<br>agcaaggaataaatcgaagagaaaaaaatgccacgtgactttgatggctaaaatcaggttatactact<br>gtacggtcccccacacataacctttaccacacaccgcacgggctggactcatcctattagaaagcgg<br>tgcagccagggccaagaaaacgcgaacgacgcagaaaaaacgtcaagcaaaaaaactgtggtgtt<br>tttttaaagcatacaatttcctgcctccctttaatgctacggggatctagcaaatgggaaaatatcacatg<br>actttggctagaaggggtgggaaaacagagattttagtcacattgtttgatttcacgtactacacgatac

		actacactacacaatacactccagtgcaatacactccagtgcaatacactccagtgcaatacactccagtgcaatacactccagtgcaatacactgcaatacactacactgtatggttccctccccgtcctcttcaggccctcgatatgctagcgaaggatcccaagcccatcgaggaaatcattcaagggcatctgcaggtctcaaatacactaagtccaaacacaaacagcactgagctaatccaagacaccaattgcatgccttctcaattatatcagtttattacacagcttataactgtgcatcttgccattcttttcgagatagccatttgattaacatgcttagctcgttctcaagccacagtaaaatggattgccttttgagtttccatcgttgtataaataggctctcattcctatagccttgctctttggtcttgtctcggtaatagtaatagctgtaagtcaggatataacactccagaaaagaaacacctaacaa**TATG**
pCgTK04	CgMT1p	**AACG**tctggaacttctgggaactcgctcccacacagaagaaatcgcaccgtgctagaccggatcagaagggatatcagcagggatcgcagtcgcggagtaacggccgtacacactctctcgtcacaactggaaagaacaaccgacgggccggatcggaacataaattgctttttcctgcccttcctttttctgatgctcatacgtgctgctctgtgcttgcagcgcaatttgtagatctgttgtacgcgtgtttctcaagcaccaacccagtcaactttgagcttaactgcctcactattaataatatccgcatctcacaaataaccaattttgctgattctgtctagtgccacttgtcacttgccagtgccagtgccagtgctcacttgccaggtttgctgcttaaagaattccgctcttcatacacatcctacactcccttaaggagggaagtacaaacaagaagtgttcgcccattctctctcacatgatgtgtaaagaaaagtgaaaatcagcaataatagcttctgactatgactatctgtattgacagcaataatagctttcgggatagcatgcaaaaaaaaatttatatataaacagaggtctttttgaaatgttagatcaatttctaattaaacattttgtagatcctacataactcatacaacaaacaacaaacacatacaaaaacaaacaca**TATG**
pCgTK05	CgTDH3p	**AACG**tcattcaacaaacaccttttctttagatcttgcttttgttattacccttactattgtctggctctgcgccattctttccactaacaatacgggtgcatggcaatcattttatgaaaatatatacacatacatctcatgataaatttcagttgtagcctcccatcaagagttagttaaaccacatgtatagctgtctctaggaataatgaaatgataatgcaggcccgagactgtattattgtgtctttggcaccttaaaaaaaagtgtcttccctccatagtgtgcacctacaaaaaatttacatacatatcccatagtgctaacaacttccttagataagacaaaactctatagtgaccaagaacagtattatcaaaattcaataacctgcaatatgacaattcccaacaaagagacaattatcaattctagaataactcttgtgcattttacactcaactgaagaggtattaatccctctcaagaagcaaacagcataataggatatgcctcccagcttcaaaataatcatataaatatgtgccctctttgcaagtaactgacatcaattgtccttgaattcattccattttaaacattatctataaagcttgaaaaaaccacagctacaagtattaattactacaggctaaagatacacagaacacatacacataacaaaacttattaacttcaaaa**TATG**
pCgTK06	CgPGK1p	**AACG**acacggctgtggtcgtcgtgacgaccacagccgtgtataataacaaaaagcagttaacaaataatagcaaacggtacacatgtgactaccacgtattgtatgctttacgggtgacaagaaaaggaaaccccgccaagatacgtgcaagacgagaataccagctttctgcttttctttcttcttcctcgatttaatggctgggggggaactgtgagattaccatgacacccgtgcacccccggactccactaggggaagcatccaggtcgggtgggagtgagagggggaatcgggcaactgctctaggagaagctctaggagaagctctaggagaagccctaggagaagcaaaggggaagcccaaagagggacaagcgcaggccactacagtaacaaagacaggaagcccaaaagatcgcaccttccagtccatcgccttcccccattgtcatatggcagtttatagcagtgtggcagtatggcagtgtggcaatgtgaagcagtgtgcaatacttccaaagtggataatgtgatgcttgcaaaaagttgggaccaccaatgctgtagagacaatgggataccctagcgaatgaagaaaaaaaggaaaacgaaaaaaatttgtatataaagcagaggcaatttttgtagcggcatgattagttgtcaattggcaagacggcatacatacatctattcgata**TATG**
pCgTK07	Core 1 UAS c-e-f	ggcgcgccCCTCCTTGGAACTGAAATTTTAGCATGTGAttaattaacttgtaatattctaatcaagctt**tataaaagagcactgttgggcgtgagtggaggcgccgg**aaaaaagcatcgaaaaaatctag
pCgTK08	Core 5 UAS c-e-f	ggcgcgccCCTCCTTGGAACTGAAATTTTAGCATGTGAttaattaacttgtaatattctaatcaagctt**tataaaagggccttggtctgaaactcctgcgtctcgcg**aaaaaagcatcgaaaaaatctag
pCgTK09	Core 8 UAS c-e-f	ggcgcgccCCTCCTTGGAACTGAAATTTTAGCATGTGAttaattaacttgtaatattctaatcaagctt**tataaaagcaatacttgggtcgacttgttatacgcgga**aaaaaagcatcgaaaaaatctag
pCgTK10	Core 9 UAS c-e-f	ggcgcgccCCTCCTTGGAACTGAAATTTTAGCATGTGAttaattaacttgtaatattctaatcaagctt**tataaaagggcgctgcgtaaggagtgctgccaggtggt**aaaaaagcatcgaaaaaatctag

pCgTK11	NLS-dCas9-Mxi1	**TATG**tctagagccccaaagaagaagagaaaagttagacccgggatggacaagaagtactccat<br>tgggctcgctatcggcacaaacagcgtcggctgggccgtcattacggacgagtacaaggtgccga<br>gcaaaaaattcaaagttctgggcaataccgatcgccacagcataaagaagaacctcattggcgccct<br>cctgttcgactccggggaaacggccgaagccacgcggctcaaaagaacagcacggcgcagatat<br>acccgcagaaagaatcggatctgctacctgcaggagatctttagtaatgagatggctaaggtggatg<br>actctttcttccataggctggaggagtcctttttggtggaggaggataaaaagcacgagcgccaccca<br>atctttggcaatatcgtggacgaggtggcgtaccatgaaaagtacccaaccatatatcatctgaggaa<br>gaagcttgtagacagtactgataaggctgacttgcggttgatctatctcgcgctggcgcatatgatcaa<br>atttcggggacacttcctcatcgagggggacctgaacccagacaacagcgatgtcgacaaactcttt<br>atccaactggttcagacttacaatcagcttttcgaagagaacccgatcaacgcatccggagttgacgc<br>caaagcaatcctgagcgctaggctgtccaaatcccggcggctcgaaaacctcatcgcacagctccc<br>tggggagaagaagaacggcctgtttggtaatcttatcgccctgtcactcgggctgacccccaacttta<br>aatctaacttcgacctggccgaagatgccaagcttcaactgagcaaagacacctacgatgatgatctc<br>gacaatctgctggcccagatcggcgaccagtacgcagaccttttttggcggcaaagaacctgtcag<br>acgccattctgctgagtgatattctgcgagtgaacacggagatcaccaaagctccgctgagcgctag<br>tatgatcaagcgctatgatgagcaccaccaagacttgactttgctgaaggcccttgtcagacagcaac<br>tgcctgagaagtacaaggaaattttcttcgatcagtctaaaaatggctacgccggatacattgacggc<br>ggagcaagccaggaggaattttacaaatttattaagcccatcttggaaaaaatggacggcaccgagg<br>agctgctggtaaagcttaacagagaagatctgttgcgcaaacagcgcactttcgacaatggaagcat<br>cccccaccagattcacctgggcgaactgcacgctatcctcaggcggcaagaggatttctacccctttt<br>tgaaagataacagggaaaagattgagaaaatcctcacatttcggataccctactatgtaggcccctc<br>gcccggggaaattccagattcgcgtggatgactcgcaaatcagaagaaaccatcactccctggaact<br>tcgaggaagtcgtggataagggggcctctgcccagtccttcatcgaaaggatgactaactttgataaa<br>aatctgcctaacgaaaaggtgcttcctaaacactctctgctgtacgagtacttcacagtttataacgagc<br>tcaccaaggtcaaatacgtcacagaagggatgagaaagccagcattcctgtctggagagcagaaga<br>aagctatcgtggacctcctcttcaagacgaaccggaaagttaccgtgaaacagctcaaagaagacta<br>tttcaaaaagattgaatgtttcgactctgttgaaatcagcggagtggaggatcgcttcaacgcatccct<br>gggaacgtatcacgatctcctgaaaatcattaaagacaaggacttcctggacaatgaggagaacga<br>ggacattcttgaggacattgtcctcacccttacgttgtttgaagatagggagatgattgaagaacgcttg<br>aaaacttacgctcatctcttcgacgacaaagtcatgaaacagctcaagaggcgccgatatacaggat<br>gggggcggctgtcaagaaaactgatcaatgggatccgagacaagcagagtggaaagacaatcctg<br>gattttcttaagtccgatggatttgccaaccggaacttcatgcagttgatccatgatgactctctcacctttt<br>aaggaggacatccagaaagcacaagtttctggccaggggggacagtcttcacgagcacatcgctaat<br>cttgcaggtagcccagctatcaaaaagggaatactgcagaccgttaaggtcgtggatgaactcgtca<br>aagtaatgggaaggcataagcccgagaatatcgttatcgagatggcccgagagaaccaaactaccc<br>agaagggacagaagaacagtagggaaaggatgaagaggattgaagagggtataaaagaactggg<br>gtcccaaatccttaaggaacacccagttgaaaacacccagcttcagaatgagaagctctacctgtact<br>acctgcagaacggcagggacatgtacgtggatcaggaactggacatcaatcggctctccgactacg<br>acgtggatgctatcgtgccccagtcttttctcaaagatgattctattgataataaagtgttgacaagatcc<br>gataaaaatagagggaagagtgataacgtcccctcagaagaagttgtcaagaaaatgaaaaattatt<br>ggcggcagctgctgaacgccaaactgatcacacaacggaagttcgataatctgactaaggctgaac<br>gaggtggcctgtctgagttggataaagccggcttcatcaaaaggcagcttgttgagacacgccagat<br>caccaagcacgtggcccaaattctcgattcacgcatgaacaccaagtacgatgaaaatgacaaactg<br>attcgagaggtgaaagttattactctgaagtctaagctggtttcagatttcagaaaggactttcagttttat<br>aaggtgagagagatcaacaattaccaccatgcgcatgatgcctacctgaatgcagtggtaggcactg<br>cacttatcaaaaaatatcccaagcttgaatctgaatttgtttacggagactataaagtgtacgatgttagg<br>aaaatgatcgcaaagtctgagcaggaaataggcaaggccaccgctaagtacttcttttacagcaatat<br>tatgaatttttcaagaccgagattacactggccaatggagagattcggaagcgaccacttatcgaaac<br>aaacggagaaacaggagaaatcgtgtgggacaagggtagggatttcgcgacagtccggaaggtc<br>ctgtccatgccgcaggtgaacatcgttaaaaagaccgaagtacagaccggaggcttctccaaggaa<br>agtatcctcccgaaaaggaacagcgacaagctgatcgcacgcaaaaaagattgggaccccaagaa<br>atacggcggattcgattctcctacagtcgcttacagtgtactggttgtggccaaagtggagaaaggga<br>agtctaaaaaactcaaaagcgtcaaggaactgctgggcatcacaatcatggagcgatcaagcttcga<br>aaaaaaccccatcgactttctcgaggcgaaaggatataaagaggtcaaaaaagacctcatcattaag<br>cttcccaagtactctctctttgagcttgaaaacggccggaaacgaatgctcgctagtgcgggcgagct

		gcagaaaggtaacgagctggcactgccctctaaatacgttaatttcttgtatctggccagccactatga<br>aaagctcaaaggttctcccgaagataatgagcagaagcagctgttcgtggaacaacacaaacacta<br>ccttgatgagatcatcgagcaaataagcgaattctccaaaagagtgatcctcgccgacgctaacctcg<br>ataaggtgctttctgcttacaataagcacagggataagcccatcagggagcaggcagaaaacattat<br>ccacttgtttactctgaccaacttgggcgcgcctgcagccttcaagtacttcgacaccaccatagaca<br>gaaagcggtacacctctacaaaggaggtcctggacgccacactgattcatcagtcaattacggggct<br>ctatgaaacaagaatcgacctctctcagctcggtggagacagcagggctgacgagggagctccca<br>agaaaaagcgcaaggtaggtagttccaagcttggcggcagcggcggcagcatggaacgtgtgag<br>aatgattaatgtgcaaaggctgttagaagccgcagagtttttagaaagaagagaaagagaatgcgaa<br>cacgggtatgccagttctttccctagcatgccctctcccagaggctaa**ATCC**

Supplementary Table 4. Primers used in this study.

Name	Sequence	Comment
SB121	GCATCGTCTCATCGGTCTCAAACGATACC AGTTACAATTAGTATTACAAT	MET3p fw (type 3)
SB122	ATGCCGTCTCAGGTCTCACATATTGTTAGG TGTTTCTTTTCT	MET3p rv (type 3)
SB124	GCATCGTCTCATCGGTCTCAAACGTCTGGA ACTTCTGGGAA	MT-Ip fw (type 3)
SB125	ATGCCGTCTCAGGTCTCACATATGTGTTTG TTTTTGTATGTG	MT-Ip rv (type 3)
SB156	GATTCTGTGGATAACCG	YTK01 entry vector seq, forward
SB157	GTTCAGAACGCTCGG	YTK01 entry vector seq, reverse
SB226	GTAGTGACTAAGGTTGGCC	For sequencing of Venus assemblies
SB202	CCCCATCTTCGTATCTTG	For sequencing of mRuby2 assemblies
SB322	GCATCGTCTCATCGGTCTCATACAAGTGCA AAGGCATTTCC	Trp1_TU_part 1 fw
SB323	ATGCCGTCTCAGACTAAGTAAGCTTCAAC GGATTCC	Trp1_TU_part 1 rv
SB324	GCATCGTCTCAAGTCTCTTTGTTCGAAGAC ACC	Trp1_TU_part 2 fw
SB325	ATGCCGTCTCAATACGTCAACACCAATTA CACCAG	Trp1_TU_part 2 rv
SB326	GCATCGTCTCAGTATCAGGTGGTGTAGAG ACTGACG	Trp1_TU_part 3 fw
SB327	ATGCCGTCTCAGGTCTCAACTCGACTAAAT TTGGTGAAGATACTTCTG	Trp1_TU_part 3 rv
RP34	AACCTCATCGCACAGCTC	For sequencing of pCgTK11
RP36	GCAAGAGGATTTCTAC	For sequencing of pCgTK11
RP39	GGCTGTCAAGAAAACTG	For sequencing of pCgTK11
RP42	CTCCACTTTGGCCAC	For sequencing of pCgTK11
RP43	GACAAGCTGATCGCAC	For sequencing of pCgTK11

RP47	TGATAGATCCAGTAATGACC	For sequencing of pCgTK11
C. glabrata Actin fw	TTACCGCTTTGGCTCCATCTTC	qPCR primer
C. glabrata Actin rev	TGTGGTGAACAATGGATGGACC	qPCR primer
C. glabrata Venus fw	ACCATGGGTAATACCAGCAGCA	qPCR primer
C. glabrata Venus rev	GGTGGTGTTCAATTAGCTGACCA	qPCR primer

Supplementary Table 5. gRNA sequences and targeted promoters.
For the target promoters 350 bp upstream of the start codon are given. gRNA targeting sites and PAM sequences are underlined and bold respectively. Note: some gRNAs target the reverse strand. The 5' UTR was extracted from the Candida genome database[10] and is given in green.

**gRNA**	**Sequence**
URA3p #1	atatacatgttagttaatat
URA3p #2	aggaaatatgatcccctta
URA3p #3	gcaagttcttgtaacttgta
URA3p #4	gcatcatcccagtagattag
URA3p #5	ttactatacaattccttaaa
ALG3p #1	attaggactttattcggtag
ALG3p #2	attttcgaaaccaaattatg
ALG3p #3	cattacacaaaaaagtaacc
ALG3p #4	gtcgttcattgcttatttta
**Promoter**	**Sequence**
URA3p	tttctatatacatgttatcagtcaaaccatacccataagaaaggtcaaggtaaatggtctacgtatgtttctgtgtaaaatcggattgttccgaaaatgactgctgataattgcaattttcttcatcatcgaaggactcttctcttaacggaattagctgaatagtcatctcatcg**cca**atattaactaacatgtatataaggttactatacaatt**cct**taaa**ggg**gatcatattt**cct**tacaagttacaagaacttgccagttctcgagaaccaattgcatcatcccagtagattag**tgg**atactccagctcaagactgaaaaaaaaaccatcaagagtacacttacat
ALG3p	gcaagacattaggactttattcggtag**agg**gcgagccactcgggtggcaagaacatgttaataatataattagtttcttagttaaaactgaaagtagctttacattacaattattttcgaaaccaaattatg**tgg**atgtcaggacttaaactacatgttagtacaaatatatctctgtgatgcatttac**ccg**gttactttttgtgtaatgcggtcgaatctagtataacttaaaa**cct**taaaataagcaatgaacgacaattgtaagtattgtgaattccattcatgagtttttaaataattgttctaaaaatataacaaaatataatacaagcattactggacattcg

3. SUPPLEMENTARY FIGURES

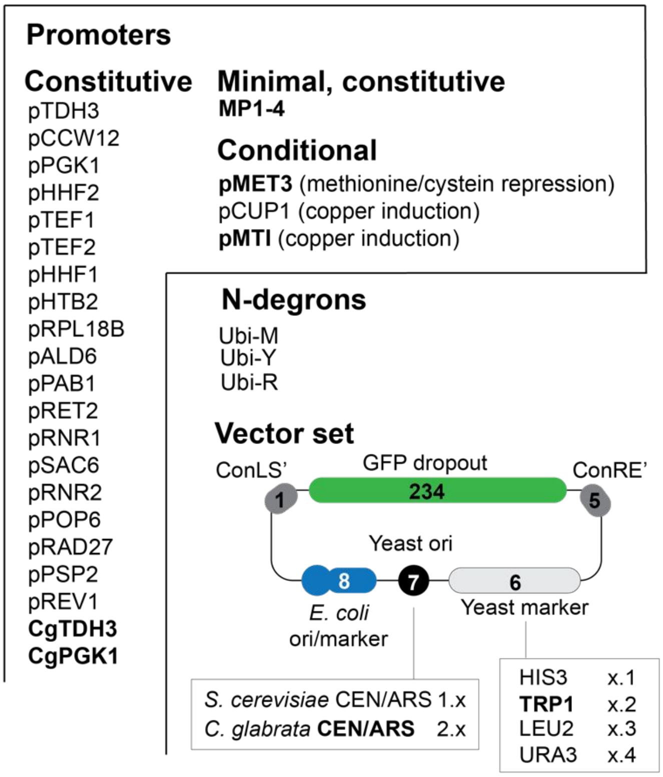

Supplementary Figure 1: Overview on the CgTK.
Overview on the YTK-parts that were characterized in *C. glabrata* and newly added parts (indicated in bold). In total, the CgTK contains 25 constitutive promoters (19 YTK, 2 from *C. glabrata* and 4 minimal promoters), three inducible/repressible promoters (copper and methionine/cysteine), three degradation tags, and nine pre-assembled vectors with different selection markers (eight auxotrophic vectors as shown here and one with a nourseotricin marker, **Supplementary Table 2**)

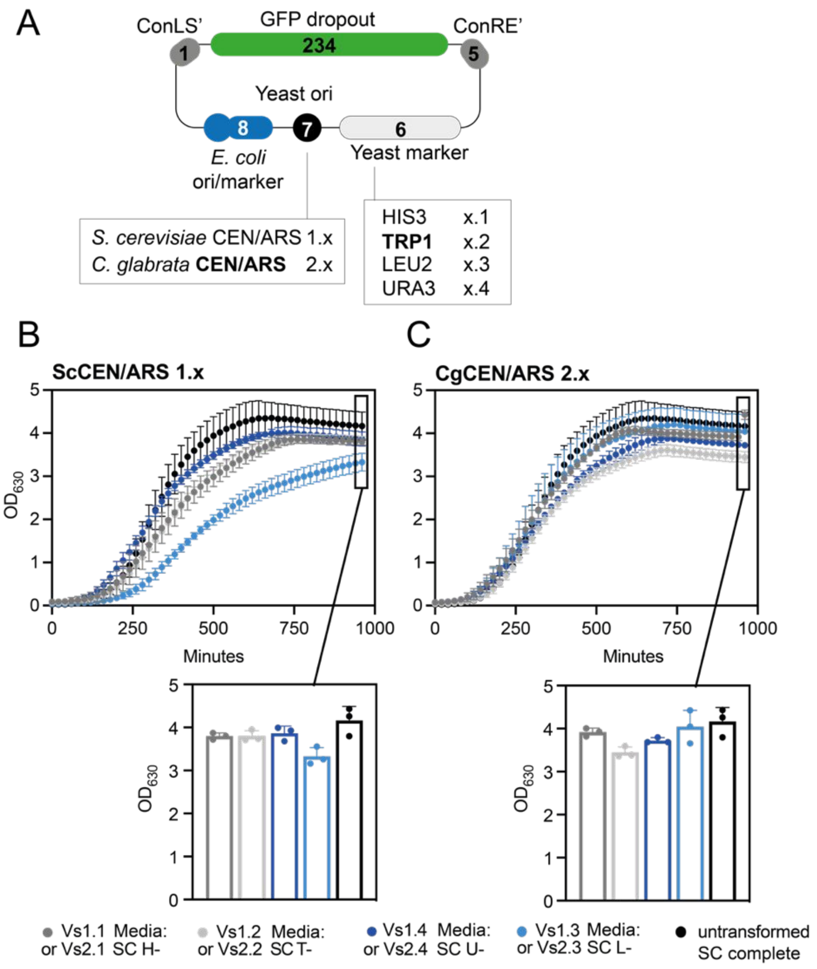

Supplementary Figure 2. Growth of *C. glabrata* transformed with empty vectors. A. Outline of the vector set featuring four auxotrophic markers and two origins of replication. **B.** Growth of *C. glabrata* transformed with ScCEN/ARS-vectors. Full growth curves and final OD_{630} is given. **C.** Growth of *C. glabrata* transformed with CgCEN/ARS-vectors. Experiments were run in biological triplicates (three transformants) and error bars represent the standard deviation.

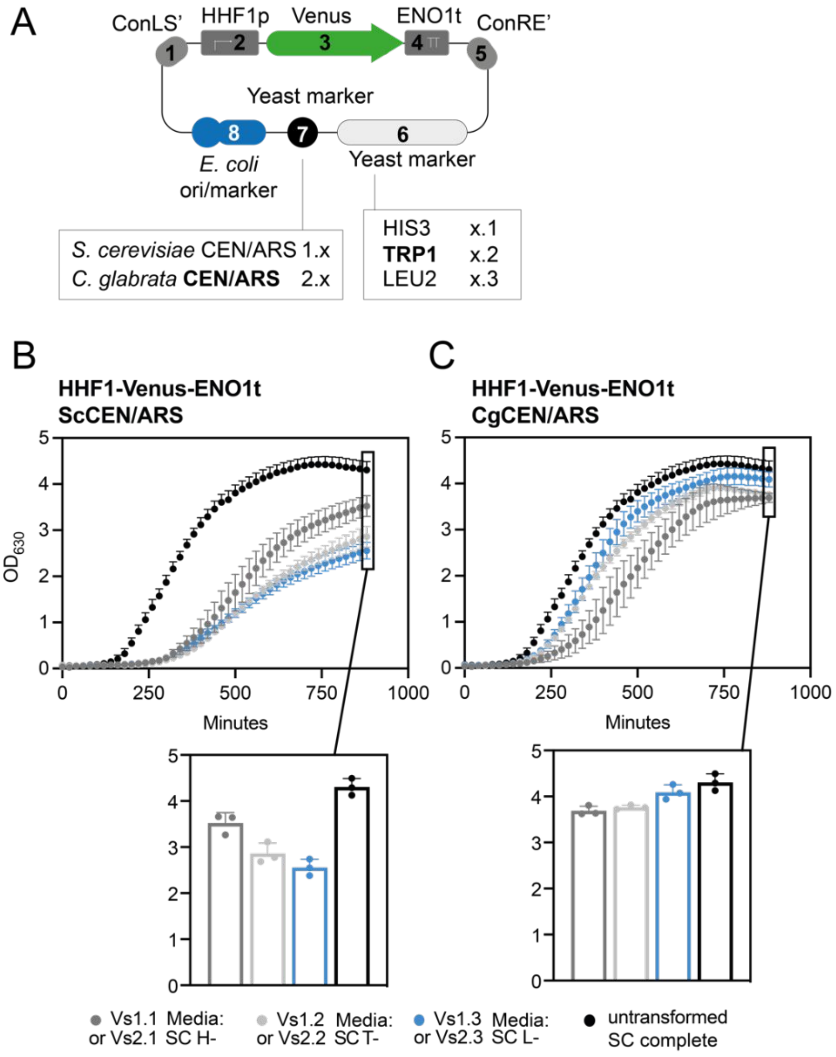

Supplementary Figure 3. Growth of *C. glabrata* transformed with Venus-expression vectors. A. Outline of the vector set featuring three auxotrophic markers and two origins of replication. **B.** Growth of *C. glabrata* transformed with ScCEN/ARS-vectors. Full growth curves and final OD_{630} is given. **C.** Growth of *C. glabrata* transformed with CgCEN/ARS-vectors. Experiments were run in biological triplicates (three transformants) and error bars represent the standard deviation.

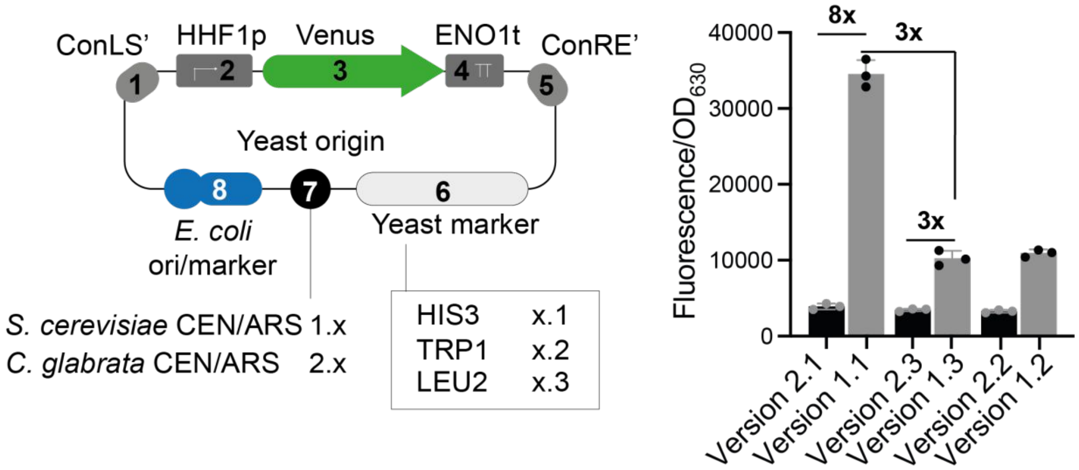

Supplementary Figure 4. Differences in expression level of Venus from the various vectors. A. Overview of used vectors. **B:** Difference in Venus expression levels. Numbers indicate fold difference between the indicated vectors. Experiments were run in biological triplicates (three transformants) and error bars represent the standard deviation.

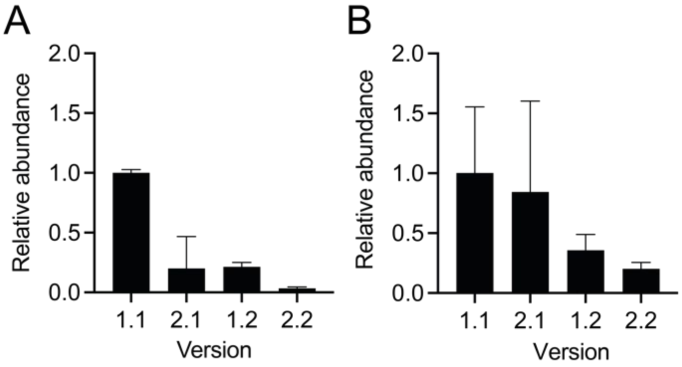

Supplementary Figure 5. qPCR analysis of relative cellular abundance of various vectors. A: qPCR on plasmid extracts **B:** qPCR based on total DNA extract. In this case plasmid copy number was normalized to actin mRNA abundance (see Methods section for both protocols).

In both (A and B), the abundance of the highest vector was set to 1 and the abundance of the other vectors is given relative to this number. Samples were measured in biological triplicates and each replicate was measured again in technical triplicate. Error bars represent standard deviation.

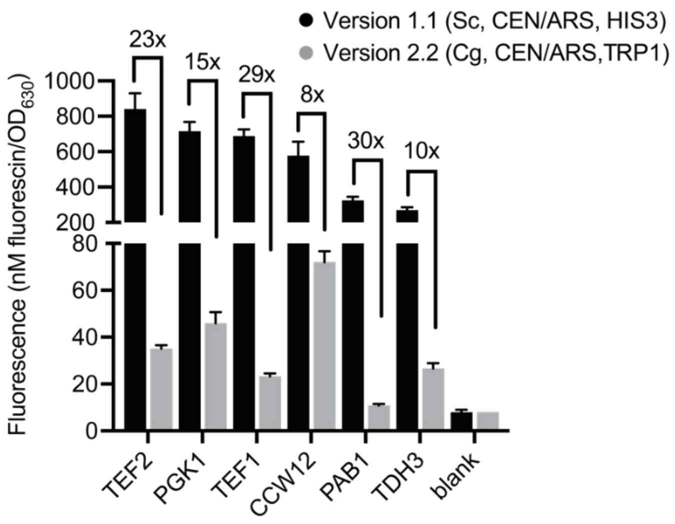

Supplementary Figure 6. Fold-difference in expression across various promoters when comparing the vector versions 1.1 and version 2.2. The same data as generated for Figure 1A and B were used. Experiments were run in biological triplicates (three transformants) and error bars represent the standard deviation.

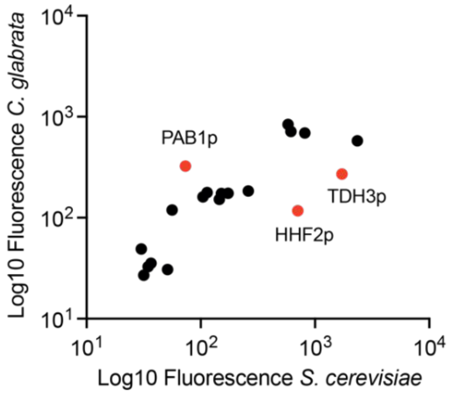

Supplementary Figure 7. **Comparison of promoter performance in *C. glabrata* and *S. cerevisiae*.** The data generated with vector version 1.1 were used for this Figure **(Figure 1A and Supplementary Figure 8A)**. Outliers are marked in red. For example, the TDH3 and HHF2 promoters ranked among the strongest promoters in *S. cerevisiae* while they showed medium (TDH3p) to low (HHF2p) expression in *C. glabrata*. On the other hand, the PAB1 promoter showed medium strength in *S. cerevisiae* and is a rather strong promoter in *C. glabrata.*

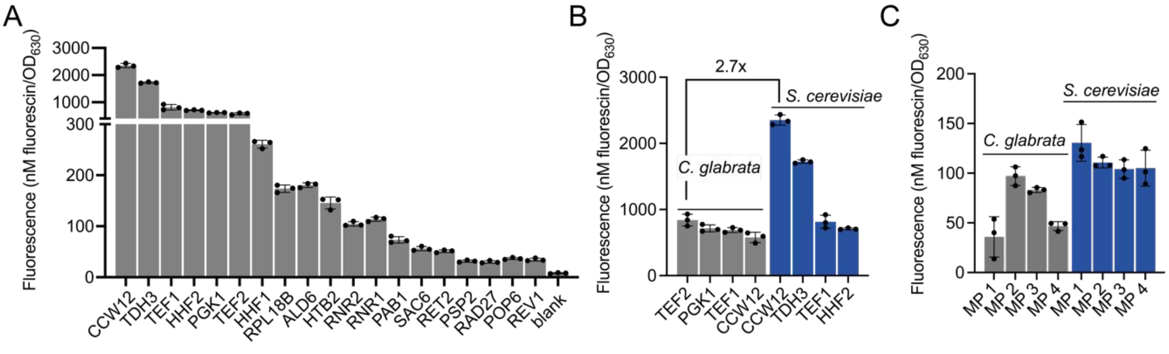

Supplementary Figure 8. Expression levels of YTK promoters and minimal promoters in *S. cerevisiae.* **A:** Performance of the 19 YTK promoters in *S. cerevisiae* using green fluorescence of the Venus protein as readout; vector version 1.1 was used for expression. **B:** The strongest promoter in *C. glabrata* (TEF2p) showed 2.7-fold lower expression than the strongest promoter in *S. cerevisiae* (CCW12p). **C:** Comparison of the expression levels resulting from the Minimal Promoters MP1-4 in *C. glabrata* and *S. cerevisiae***.** Experiments were run in biological triplicates (three transformants) and error bars represent the standard deviation. For all experiments, fluorescence measurements were taken after 16 hours of growth (see methods section).

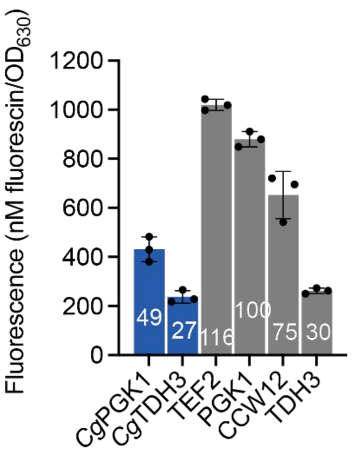

Supplementary Figure 9. **Comparison of *C. glabrata* derived TDH3 and PGK1 promoters with the strongest YTK-derived promoters.** Data were generated in vector version 1.1 using green fluorescence of the Venus protein as readout. Experiments were run in biological triplicates (three transformants) and error bars represent the standard deviation. For all experiments, fluorescence measurements were taken after 16 hours of growth (see methods section).

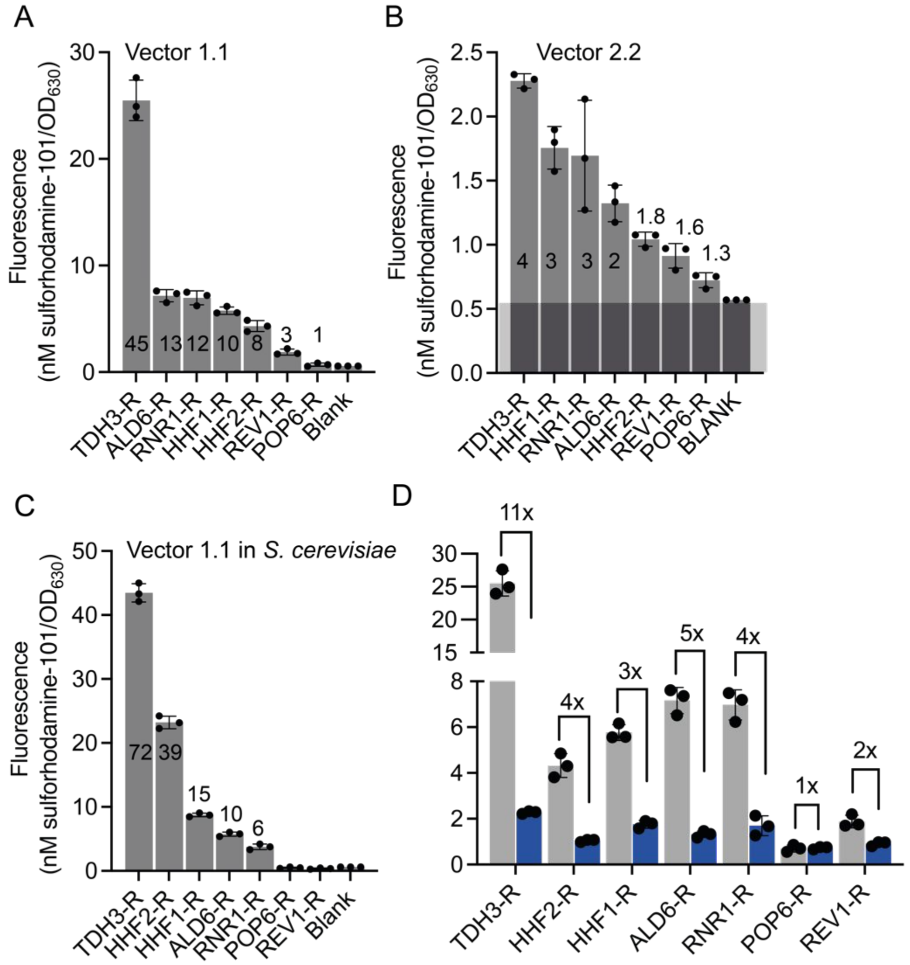

Supplementary Figure 10. Performance of seven YTK promoters in *C. glabrata and S. cerevisiae* using red fluorescence (mRuby2) as readout. A and B: Performance of the seven YTK promoters in *C. glabrata* using two different vectors; version 1.1 (A) and 2.2 (B). The numbers indicate the fold-change in fluorescence over background, where background is defined as the autofluorescence of *C. glabrata* cells not carrying a plasmid. Note: The Y-axis shows a different scale. **C:** Performance of the same seven YTK promoters in *S. cerevisiae* **D:** Fold difference in expression between vector version 1.1 and 2.2 in *C. glabrata*. Data from panels A and B were used. All arbitrary fluorescence values were normalized to the calibrant dye sulforhodamine-101.[6] All experiments were run in biological triplicates (three transformants) and error bars represent the standard deviation. For all experiments, fluorescence measurements were taken after 16 hours of growth (see methods section).

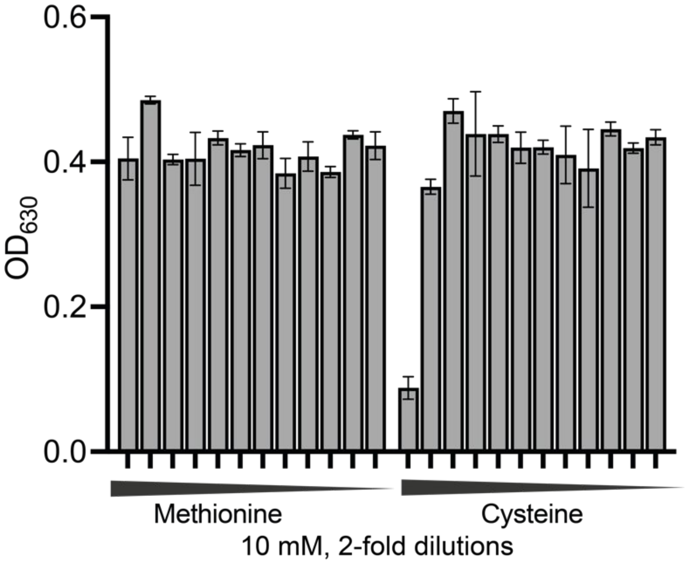

Supplementary Figure 11. Growth of *C. glabrata* in the presence of up to 10 mM methionine and cysteine. The final OD_{630} after 24 hours of growth is shown. Experiments were run in biological triplicates (three transformants) and error bars represent the standard deviation.

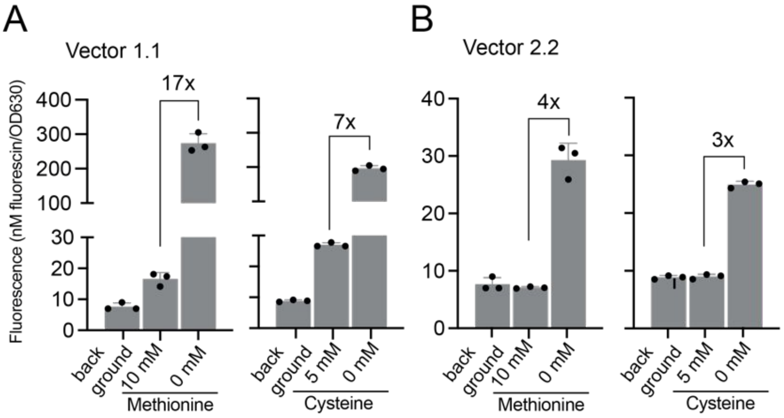

Supplementary Figure 12. ON and OFF state of Met3 promoter. Fold-change difference in expression level between the "ON" and the "OFF" state of the MET3 promoter in vector version 1.1 (A) and 2.2 (B) when induced with 10 mM methionine or 5 mM cysteine. The "OFF" state is also compared to the background autofluorescence of cells without a plasmid (blank). Experiments were run in biological triplicates (three transformants) and error bars represent the standard deviation. For all experiments, fluorescence measurements were taken after 16 hours of growth (see methods section).

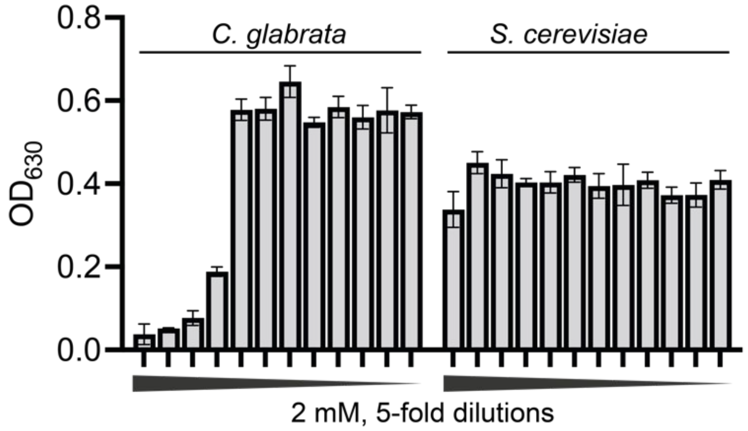

Supplementary Figure 13. Growth of *C. glabrata* and *S. cerevisiae* in the presence of up to 2 mM $CuSO_4$. The final OD_{630} after 24 hours of growth is shown. Experiments were run in biological triplicates (three transformants) and error bars represent the standard deviation.

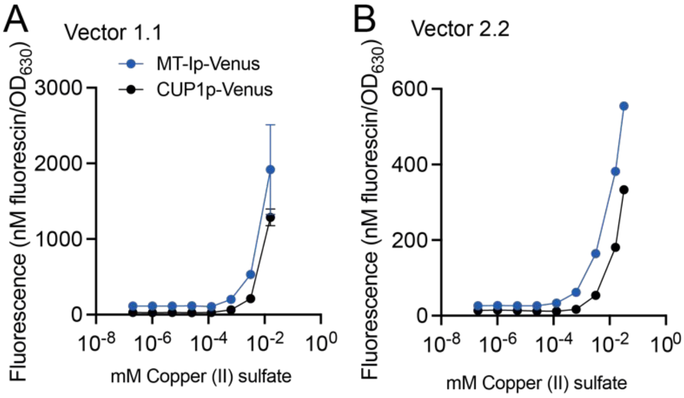

Supplementary Figure 14. Performance of the MT-I and the CUP1 promoters in vector version 1.1 (A) and 2.2 (B) when grown in the presence of increasing concentrations of $CuSO_4$ (starting from 2 mM $CuSO_4$, five-fold dilutions were measured). Note: *C. glabrata's* growth is (partly) inhibited by copper concentration above 16 µM **(Supplementary Figure 13).** More resolved data on basal expression in the absence of $CuSO_4$ can be found in **Supplementary Figure 17.** Experiments were run in biological triplicates (three transformants) and error bars represent the standard deviation. For all experiments, fluorescence measurements were taken after 16 hours of growth (see methods section).

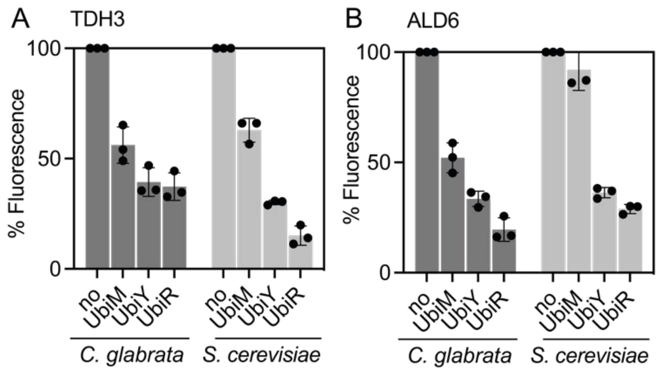

Supplementary Figure 15. Performance of the protein degradation tags Ubi-M, Ubi-Y, and Ubi-R in *C. glabrata* in comparison to *S. cerevisiae.* Experiments were performed with two promoters (*TDH3*, **A** and *ALD6*, **B**) in vector version 1.1. Experiments were run in biological triplicates (three transformants) and error bars represent the standard deviation. For all experiments, fluorescence measurements were taken after 16 hours of growth (see methods section).

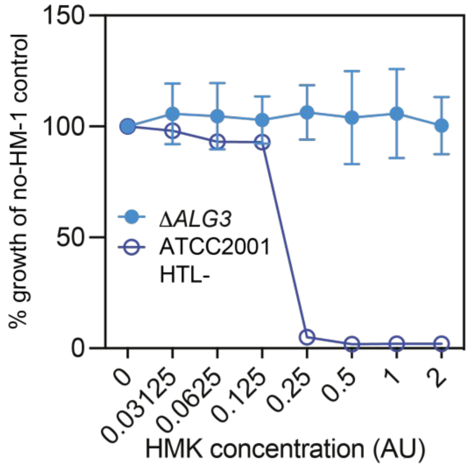

Supplementary Figure 16. Resistance of *C. glabrata* ATCC 2001 HTL$^-$ DeltaRHK1/ALG3 to HM-1. Cells were grown for 24 hours in the presence of increasing concentrations of HM-1. The final OD_{630} was normalized to the OD of cells grown in the absence of HM-1. Experiments were run in technical triplicates and error bars represent the standard deviation, SD for ATCC2001 HTL$^-$ is smaller than the size of the circles.

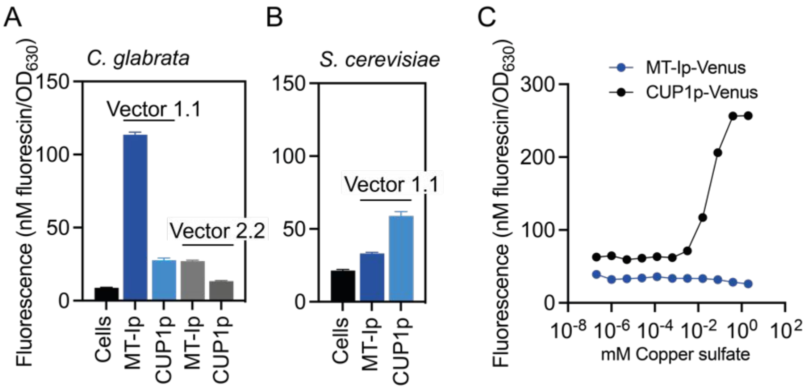

Supplementary Figure 17. A-B. Basal expression from the thwo copper inducible promoters when cloned into vector version 1.1 or 2.2 in *C. glabrata* **(A)** and *S. cerevisiae* **(B)**. **C:** Performance of the two copper inducible promoters in *S. cerevisiae*. Experiments were run in biological triplicates (three transformants) and error bars represent the standard deviation. For all experiments, fluorescence measurements were taken after 16 hours of growth (see methods section).

4. NOTES

Note 1: Growth burden caused by ScCEN/ARS Venus expression vectors. We noticed while the CgCEN/ARS vectors (containing the Venus transcriptional unit) showed very minor growth differences **(Supplementary Figure 3C)**, the ScCEN/ARS showed longer lag phases and reached only 82% (version 1.1, ScCEN/ARS, HIS3), 66% (version 1.2, ScCEN/ARS, TRP1) and 59% (version 1.3, ScCEN/ARS, LEU2) of the final OD_{630} of the untransformed control **(Supplementary Figure 3B)**.

Note 2: Performance of seven YTK promoters when driving the red fluorescent protein mRuby. Similar to green fluorescence, also for the red fluorescence vector version 1.1 showed higher expression than 2.2 (between 11 and 2-fold difference depending on promoter **(Supplementary Figure 10A, B and D)**. Vector version 1.1 yielded a 44-fold expression over background with the strongest promoter tested (the medium-level TDH3 promoter) and vector version 2.2 a 4-fold expression level over background for the same promoter. Overall, mRuby expression yielded about 10-fold less fluorescence when compared to Venus expression, measured and compared in nM calibrant dye. The lower fluorescence output with mRuby2, when compared to Venus, was also observed in *S. cerevisiae* BY4741, both in the original YTK data set[5] and in our data set **(Supplementary Figure 10C)**.

Note 3: Design of minimal promoters and potential for optimization.
The minimal promoters MP1-4 are based on the designed described by Redden *et al.*[8] They feature a short length (~129 bp versus the regular 700 bp of YTK promoters), and modularity. Various core promoters of 30 bp can be combined with constitutive or inducible upstream activating sequences (UAS) of 10 bp. Here we used the core promoters 1, 5, 8 and 9 - which had shown different strengths in *S. cerevisiae* - combined with the UASs E, F, and C. The sequences of each promoter are listed in **Supplementary Table 3.** MP1-4 showed low to medium expression strength in *C. glabrata*. Interestingly, also expression levels in *S. cerevisiae* were medium **(Supplementary Figure 8C),** although reported expression levels of these promoters should be close to the strongest promoters in *S. cerevisiae*.[8] It has been shown before that the YTK Golden Gate design leaves a BglII seam between the promoter and the ORF which is suboptimal for expression strength. This might explain why the MP design did not achieve the reported high expression levels in this specific set-up.[8] Optimization could thus be achieved by designing better Golden Gate seams.[11]

Note 4. Performance of $CuSO_4$-inducible promoters in *C. glabrata* and *S. cerevisiae*.
First, we noticed that *C. glabrata* cells were much more sensitive to $CuSO_4$ than *S. cerevisiae* cells **(Supplementary Figure 13**). For induction, we used 5-fold dilutions starting from 2 mM $CuSO_4$ and *C. glabrata* cells would only grow at concentrations lower than 16 μM (though reaching lower final OD_{630}) and would only reach full OD_{630} at concentrations lower than 3 μM. In contrast, *S. cerevisiae* cells reached full OD_{630} at the highest tested concentration of 2 mM $CuSO_4$. Still, *C. glabrata* cells seemed metabolically active and produced high levels of Venus protein at concentrations above 16 μM. For comparison*,* we also characterized both promoters in *S. cerevisiae* **(Supplementary Figure 17)**. Interestingly, the MT-1 promoter was not functional in *S. cerevisiae* **(Supplementary Figure 17C).**

Note 5: Degradation tags.
The YTK-derived degradation tags UBI-M (weak), UBI-Y (medium) and UBI-R (strong) were fused to the Venus protein cloned under the control of the TDH3 and ALD6 promoters. As before, expression levels were measured in bulk and normalized to fluorescein. **Figure 1G** shows that all three degradation tags were functional in *C. glabrata* reducing the fluorescence in cells with a TDH3-driven degradation-tagged Venus to 56% (UBI-M), 39% (UBI-Y), and 37% (UBI-R) of the untagged Venus levels. For the ALD6p-driven degradation-tagged Venus, fluorescence was reduced to 52% (UBI-M), 33% (UBI-Y), and 20% (UBI-R). In comparison, in *S. cerevisiae* the tagging led to a remaining fluorescence of 63% (UBI-M), 30% (UBI-Y) and 15% (UBI-R) for a TDH3p-driven degradation-tagged Venus protein and

to 92% (UBI-M), 36% (UBI-Y) and 23% (UBI-R) for an ALD6p-driven degradation-tagged Venus protein **(Supplementary Figure 15).**

Note 6: Composition of the CRISPRi system.
We first cloned a BsaI/BsmBI restriction site-free dCas9-Mxi1 construct as a level 0 vector and verified that this recoded fusion would be functional by testing it in *S. cerevisiae* with an established gRNA/promoter pair.[12] We then cloned the dCas9-Mxi1 fusion protein under the control of an HHF1 promoter and ENO1 terminator into vector version 1.3. The gRNAs were expressed from a second vector, version 2.2. The CRISPRi system had been cloned and tested before finding out that vector version 1.3 caused a growth burden. Vector 1.3 worked well for our proof of concept, but in the future one can consider using vector 1.1 for expressing the repressor system.
Further, we used a two-vector system instead of an integrated one-vector system for two reasons:
1. It keeps the gRNA library plasmid and the dCas9 system separate and will allow to expand it to a CRISPRa system re-cloning the library.
2. The transformation efficiency of the dCas9-Mxi1 plasmid was 2 orders of magnitude lower when compared to other plasmids, as such we had concerns about the efficiency of future library transformations in a one-vector system.

5. REFERENCES

(1) Baker Brachmann, C.; Davies, A.; Cost, G. J.; Caputo, E.; Li, J.; Hieter, P.; Boeke, J. D. Designer Deletion Strains Derived FromSaccharomyces Cerevisiae S288C: A Useful Set of Strains and Plasmids for PCR-Mediated Gene Disruption and Other Applications. *Yeast* **1998**, *14* (2), 115–132.

(2) Schwarzmüller, T.; Ma, B.; Hiller, E.; Istel, F.; Tscherner, M.; Brunke, S.; Ames, L.; Firon, A.; Green, B.; Cabral, V.; Marcet-Houben, M.; Jacobsen, I. D.; Quintin, J.; Seider, K.; Frohner, I.; Glaser, W.; Jungwirth, H.; Bachellier-Bassi, S.; Chauvel, M.; Zeidler, U.; Ferrandon, D.; Gabaldón, T.; Hube, B.; d'Enfert, C.; Rupp, S.; Cormack, B.; Haynes, K.; Kuchler, K. Systematic Phenotyping of a Large-Scale Candida Glabrata Deletion Collection Reveals Novel Antifungal Tolerance Genes. *PLoS Pathog.* **2014**, *10* (6), e1004211.

(3) Kaiser, C.; Michaelis, S.; Mitchell, A. Methods in Yeast Genetics. **1994**.

(4) Güldener, U.; Heck, S.; Fiedler, T.; Beinhauer, J.; Hegemann, J. H. A New Efficient Gene Disruption Cassette for Repeated Use in Budding Yeast. *Nucleic Acids Res.* **1996**, *24* (13),

(5) Lee, M. E.; DeLoache, W. C.; Cervantes, B.; Dueber, J. E. A Highly Characterized Yeast Toolkit for Modular, Multipart Assembly. *ACS Synth. Biol.* **2015**, *4* (9), 975–986.

(6) Beal, J.; Telmer, C. A.; Vignoni, A.; Boada, Y.; Baldwin, G. S.; Hallett, L.; Lee, T.; Selvarajah, V.; Billerbeck, S.; Brown, B.; Cai, G.; Cai, L.; Eisenstein, E.; Kiga, D.; Ross, D.; Alperovich, N.; Sprent, N.; Thompson, J.; Young, E. M.; Endy, D.; Haddock-Angelli, T. Multicolor Plate Reader Fluorescence Calibration. *Synth. Biol.* **2022**, *7* (1), 1–9.

(7) Zordan, R. E.; Ren, Y.; Pan, S. J.; Rotondo, G.; Iluore, A. D. L. P.; Cormack, B. P. Expression Plasmids for Use in Candida Glabrata. *G3 Genes, Genomes, Genet.* **2013**, *3* (9), 1675–1686.

(8) Redden, H.; Alper, H. S. The Development and Characterization of Synthetic Minimal Yeast Promoters. *Nat. Commun.* **2015**, *6* (1), 1–9. https://doi.org/10.1038/ncomms8810.

(9) Smith, J. D.; Suresh, S.; Schlecht, U.; Wu, M.; Wagih, O.; Peltz, G.; Davis, R. W.; Steinmetz, L. M.; Parts, L.; St.Onge, R. P. Quantitative CRISPR Interference Screens in Yeast Identify Chemical-Genetic Interactions and New Rules for Guide RNA Design. *Genome Biol.* **2016**, *17* (1).

(10) Skrzypek, M. S.; Binkley, J.; Binkley, G.; Miyasato, S. R.; Simison, M.; Sherlock, G. The Candida Genome Database (CGD): Incorporation of Assembly 22, Systematic Identifiers and Visualization of High Throughput Sequencing Data. *Nucleic Acids Res.* **2017**, *45* (D1), D592–D596.

(11) Otto, M.; Skrekas, C.; Gossing, M.; Gustafsson, J.; Siewers, V.; David, F. Expansion of the

Yeast Modular Cloning Toolkit for CRISPR-Based Applications, Genomic Integrations and Combinatorial Libraries. *ACS Synth. Biol.* **2021**, *10* (12), 3461–3474.
(12) McCarty, N. S.; Shaw, W. M.; Ellis, T.; Ledesma-Amaro, R. Rapid Assembly of GRNA Arrays via Modular Cloning in Yeast. *ACS Synth. Biol.* **2019**, *8* (4), 906–910.
